# Developmental biology of *Streptomyces* from the perspective of 100 actinobacterial genome sequences

**DOI:** 10.1111/1574-6976.12047

**Published:** 2013-11-19

**Authors:** Govind Chandra, Keith F Chater

**Affiliations:** John Innes Centre, Norwich Research ParkNorwich, UK

**Keywords:** mycelial growth, polar growth, cell division, sporulation, nitric oxide, mycothiol

## Abstract

To illuminate the evolution and mechanisms of actinobacterial complexity, we evaluate the distribution and origins of known *Streptomyces* developmental genes and the developmental significance of actinobacteria-specific genes. As an aid, we developed the Actinoblast database of reciprocal blastp best hits between the *Streptomyces coelicolor* genome and more than 100 other actinobacterial genomes (http://streptomyces.org.uk/actinoblast/). We suggest that the emergence of morphological complexity was underpinned by special features of early actinobacteria, such as polar growth and the coupled participation of regulatory Wbl proteins and the redox-protecting thiol mycothiol in transducing a transient nitric oxide signal generated during physiologically stressful growth transitions. It seems that some cell growth and division proteins of early actinobacteria have acquired greater importance for sporulation of complex actinobacteria than for mycelial growth, in which septa are infrequent and not associated with complete cell separation. The acquisition of extracellular proteins with structural roles, a highly regulated extracellular protease cascade, and additional regulatory genes allowed early actinobacterial stationary phase processes to be redeployed in the emergence of aerial hyphae from mycelial mats and in the formation of spore chains. These extracellular proteins may have contributed to speciation. Simpler members of morphologically diverse clades have lost some developmental genes.

## Introduction

Bacteria in the ancient phylum *Actinobacteria* have extraordinary diversity of function and form. They include pathogens of humans and other mammals (the agents of tuberculosis, leprosy, mycetomas, diphtheria, Whipple's disease, and skin, oral and vaginal infections of humans) and plants (potato scab, ratoon stunting disease of sugarcane); major agents of symbiotic nitrogen fixation (*Frankia*); industrially important producers of amino acids (*Corynebacterium glutamicum*); genera such as *Streptomyces*, *Micromonospora*, *Saccharopolyspora* and *Actinoplanes* that are the richest natural source of antibiotics and other secondary metabolites; probiotic bifidobacteria; and agents of bioremediation, notably rhodococci (Ventura *et al*., 2007). There is also growing interest in their frequent occurrence as plant endophytes and arthropod exosymbionts (Seipke *et al*., 2012).

**Fig. 1:**
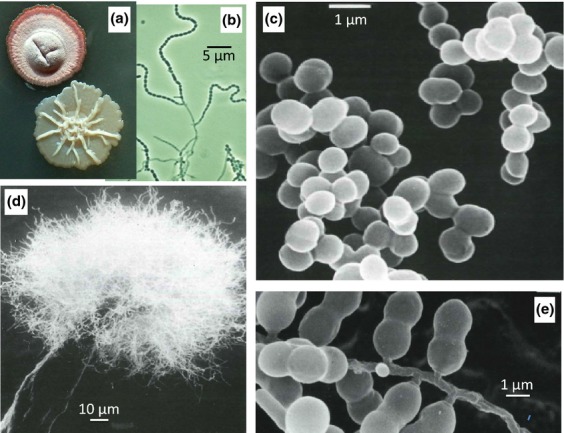
Morphological diversity of actinobacteria. (a) Colonies of *Streptomyces coelicolor* A3(2) wild type (upper) and *bldA* mutant (lower). (b) Phase contrast image of sporulating mycelium of *S. coelicolor*. (c) *Micrococcus luteus*. (d) *Actinosynemma mirum*. (e) *Microbispora rosea*. Images [c–e] are scanning electron micrographs taken from Miyadoh (1997), with permission.

Actinobacteria are Gram-positive bacteria that typically have a high G + C content in their DNA. They range from simple cocci to the various complex mycelial forms found in some of the *Actinomycetales* order (Fig. 1). This morphological diversity is spectacularly illustrated in the ‘Atlas of Actinomycetes’ (Miyadoh, 1997). Mycelial organisms present particular problems for growth and development: their hyphae are intrinsically nonsymmetrical; special mechanisms must be needed to permit and control branching; and they must have some phase of fragmentation that permits dispersal. Often, the fragmentation of actinomycete hyphae leads to the formation of dessication-resistant spores, of a general type distinct from the endospores formed inside ‘mother cells’ of *Bacillus* spp. and other firmicute bacteria: they are formed directly by cell division from multigenomic hyphal compartments, followed by changes in the cell wall to permit rounding and thickening of the spore wall and the acquisition of resistance properties. These ‘exospores’ appear in or on a considerable variety of specialised morphological structures, including short hyphal side branches, large sporangia and specialised aerial hyphae that turn into long spore chains. In some genera, although not in *Streptomyces*, spores may be motile.

Streptomycetes, the central subject of this article, are the most extensively studied mycelial actinobacteria. They are sporulating organisms whose considerable morphological complexity is interlinked with an extraordinary ability to make diverse secondary metabolites (Chater, 2011; Liu *et al*., 2013a). Two or three days after a spore germinates on agar media, the biomass-accumulating vegetative or substrate mycelium of the colony becomes covered with a fuzzy white aerial mycelium. The individual aerial hyphae grow to give rise to long unbranched tip cells often containing more than 50 copies of the genome. The tip cells are then divided into multiple prespore compartments by sporulation septation, during which synchronously assembled and regularly spaced FtsZ rings lead septal ingrowth. During sporulation septation, the uncondensed mass of chromosomes partitions into nucleoids, so that each prespore compartment contains a single copy of the genome. The change of cylindrical prespore compartments into ovoid spores involves remodelling and thickening of the cell wall, while inside the developing spore further changes contribute to the onset of dormancy, including chromosome condensation.

Three model species have provided nearly all the available experimental information about the molecular basis of the morphological development of streptomycetes. The most widely studied of these is the genetically amenable *S. coelicolor* A3(2) (Hopwood, 2007), while *S. griseus* (one of the first streptomycetes to be used as the source of a major antibiotic, streptomycin) has been particularly intensively studied for its production of, and responsiveness to, a hormone-like developmental signalling molecule, A-factor (Horinouchi, 2002, 2007). The third model species, *S. venezuelae*, an early industrial producer of chloramphenicol, has recently been taken up as a developmental model, because it sporulates rapidly, synchronously and comprehensively in submerged culture, in contrast to most streptomycetes, which sporulate gradually and nonsynchronously and do not form spores in submerged culture (Flärdh & Buttner, 2009; Bibb *et al*., 2012). This makes *S. venezuelae* especially suitable for sensitive biochemical, cytological and molecular studies of consecutive developmental states. The genomes of all three species have been sequenced (Bentley *et al*., 2002; Ohnishi *et al*., 2008; FR845719: for annotated presentation, see http://strepdb.streptomyces.org.uk), along with those of numerous other members of the genus.

A previous comparative genomic survey of actinobacteria (Ventura *et al*., 2007) was based on 21 sequences, encompassing 10 genera, and with many gaps in its phylogenetic coverage. When we began the analysis leading to this article in April 2011, about 100 further actinobacterial genomes had been sequenced and annotated to a level that made productive comparative analysis possible. The number had increased to 157 complete sequences and 474 in progress, in a recent and comprehensive review on the genome-based phylogeny of actinobacteria (Gao & Gupta, 2012). That review extended earlier work in which 28 ‘signature proteins’ peculiar to, and near-universal among, actinobacteria were identified, along with a further 48 peculiar to, and near-universal among, actinomycetes (Gao *et al*., 2006). These proteins form an important background to this review, and we summarise them in Table 1, using *Streptomyces coelicolor* gene designations (*SCO* numbers) as the key instead of those originally used (mainly from *Mycobacterium leprae*).

**Table 1:** Conserved actinobacterial signature proteins/genes identified by Gao *et al*. (2006) and Gao & Gupta (2012)†. (A) The 26 most frequent actinobacterial signature proteins include at least six with likely developmental roles (asterisks). (B) Seven actinomycete signature proteins referred to in the text‡

SCO number	ML number	Comments such as gene or protein name, function, conserved linkage, references, etc.
(A)		
5199	0642	Often next to conserved gene for ‘epimerase/dehydratase’. Similar to SCO3407 (25% identity over 336 aa overlap), which is also very widespread and actinospecific, but is not listed in Gao *et al*. (2006). *SCO3407* is neighboured by *SCO3408* (= *ML00211*, actinospecific, widely conserved, predicted D-ala, D-ala carboxypeptidase, PBP4 class, similar to *dacB* of *E. coli*) and by a cluster conserved even in *B. subtilis* (*SCO3406*, possible MesJ-like cell division-associated ATPase; *SCO3405*, probable hypoxanthine phosphoribosyl transferase; *SCO3405*, FtsH2, ATP-dependent protease)
1997*	1009	Closely similar to ParJ. Function unknown, but structure established (Gao *et al*., 2009). May perhaps interact with ParA or the ubiquitous ParA2 (= SCO1772). Absent from nonactinomycete actinobacteria and from two actinomycetes, *Trophyrema whipplei* and *Saccharopolyspora erythraea*.
5869	1029	DUF3710 domain; probably cotranscribed with *SCO5868* (3nt in between; *dut*, probable deoxyuridine 5′-triphosphate nucleotidohydrolase) and *SCO5867* (phenylacetic acid thioesterase, Paa1). Linkage with *dut* conserved throughout actinomycetes
1662*	1306	*parJ*. ParJ interacts with ParA (Ditkowski *et al*., 2010)
3034*	0760	*whiB*, developmental regulatory gene (Fowler-Goldsworthy *et al*., 2011)
5240*	0804	*wblE*, encodes WhiB-like protein of uncertain role, possibly essential in streptomycetes but not in *C. glutamicum* (Kim *et al*., 2005; Fowler-Goldsworthy *et al*., 2011)
2196	0857	234 aa, probable integral membrane protein
2169	0869	251 aa, DUF3034, probable integral membrane protein
2947	1016	97 aa, DUF3039
5864	1026	98 aa; note conserved linkage of *SCO5864* and *5869*, and *ML1026* and *1029*
1381	2073	228 aa; present in all actinobacteria except *Acidimicrobium ferrooxidans* and *Coriobacteriales*. Removed by Gao & Gupta (2012)
5855	2137	252 aa, DUF3071
4088	2204	84 aa, DUF3073
3854*	0013	*crgA* (*whiP* in *S. avermitilis*), 84-aa membrane protein, septation inhibitor, absent from nonactinomycete actinobacteria (Del Sol *et al*., 2003, 2006; Plocinski *et al*., 2011, 2012)
3872	0007	185 aa, DUF3566, invariably very close to *oriC*
1938	0580	*opcA*; assembly of glucose-6-phosphate dehydrogenase (also in cyanobacteria: Hagen & Meeks, 2001; and next to the *zwf2* gene); a less widely occurring paralogue, *SCO6660*, is downstream of *zwf1* in *S. coelicolor*
2078	0921	94 aa, possible transmembrane protein, invariably next to *divIVA*
1421	1439	*rpbA,* RNA polymerase-binding protein (Tabib-Salazar *et al*., 2013; Bortoluzzi *et al*., 2013; note that ML1439 was listed twice by Gao *et al*. (2006))
5601	1610	102aa, DUF2469, conserved linkage with *SCO5602*
4084	2207	437 aa. Note conserved linkage of *SCO4084* and *4088*, and *ML2207* and *2204*
3095*	0256	*divIC;* part of cell division apparatus, interacts with FtsL (Bennett *et al*., 2007; sequence divergence of DivIC orthologues in other bacteria took them beyond the threshold adopted by Gao *et al*., 2006; but they were removed by Gao & Gupta, 2012)
3011	0775	*lpqB*/Putative lipoprotein
3031	0761	117 aa, DUF1025. Note conserved linkage of *SCO3031* and another signature gene, *SCO3034* (*whiB*)
5169	0814	94 aa, DUF3107, possible ATP-binding protein
2370	1649	159 aa, DUF3052, invariably next to gene for possible thiol-specific antioxidant protein
4330	2142	308 aa, DUF3027
(B)		
3375	0234	lsr2/HNS-like DNA-bridging protein, iron-regulated in *M. tuberculosis* (Gordon *et al*., 2010)
2097	0904	135 aa, DUF3040, part of spore wall-synthesising complex (Kleinschnitz *et al*., 2011)
4179	2200	191 aa, cd07821, likely nitrobindin. NO or fatty acid-binding protein domain, structure known for *M. tuberculosis* (Shepard *et al*., 2007), conserved synteny with adjacent *fur* homologue
1480	0540	107 aa, nucleoid-binding protein sIHF (Yang *et al*., 2012; Swiercz *et al*., 2013)
1664	1300	265 aa, generally very close to *mshC* gene for mycothiol biosynthesis
3097	2030	*rpfC*/RPF, secreted protein, peptidoglycan binding, several paralogues
4205	2442	168 aa, DUF2596, downstream of and overlapping *mshA*

† The gene identifiers listed by Gao *et al*. (2006) were for the *Mycobacterium leprae* genome. Here, we have listed *S. coelicolor* orthologues as defined by reciprocal best-hit BLASTP analysis. The function descriptions are based on the cited papers where given, but where no reference is given, the commentary is derived from synteny and conserved domain analysis carried out for this review, using StrepDB (http://strepdb.streptomyces.org.uk).

‡ The remaining 39 actinomycete signature genes identified by Gao *et al*. (2006) were as follows (*M. leprae, L. xyli* or *T. fusca* designations given in brackets after *SCO* equivalent): *SCO* numbers: *0908* (*Tfu_0365*), *1372* (*Lxx16410*), *1383* (*ML2075*), *1437* (*ML0561*), *1653* (*ML1312*), *1665* (*ML1299*), *1929* (*ML0589*), *2105* (*ML0898*), *2153* (*ML2446*), *2154* (*ML0876*), *2197* (*Lxx10090*), *2391* (*ML1781*), *2460* (*Tfu_1340*), *2557* (*Lxx08190*), *2643* (*ML1485*), *2893* (*ML0169*), *2915* (*ML1166*), *2916* (*ML1165*), *3016* (*Tfu_2498*), *3030* (*ML0762*), *3576* (*Lxx03620*), *3647* (*ML0284*), *3822* (*ML0115*), *3902* (*ML2687*), *4043* (*Tfu_0030*), *4287* (*ML1927*), *4579* (*ML2064*), *4590* (*Tfu_1240*), *5145* (*ML1067)*, *5167* (*Tfu_0515*), *5173* (*ML0816*), *5414* (*ML1176*), *5493* (*ML1706*), *5697* (*Tfu_0751*), *5766* (*ML0986*), *5866* (*ML1027*), *6030* (*ML1041*). One (*ML2428A*) was similar to *SCO3327*, but did not give a reciprocal blastp best hit, and another (*ML0899*) was absent from *S. coelicolor*, but present in *S. avermitilis* (*SAV1313*) and many other streptomycetes.

Our aim in this article is to combine comparative genomics, knowledge about *Streptomyces* development and growing information about gene function gleaned from other actinobacteria, particularly from the intensive focus of many researchers on the globally important pathogen *Mycobacterium tuberculosis*, to address several questions: What are the evolutionary origins of genes important for *Streptomyces* sporulation? Are the mechanisms leading to sporulation widely homologous in phylogenetically diverse actinobacteria, or did they evolve independently? Does the developmental process contribute to speciation? Are today's simple actinobacterial species primitive, or are they degenerate descendants of morphologically much more complex ancestors? What gave the ancestral ur-actinobacterium the potential for such morphological complexity in its modern descendants? And can studies of the development of complex actinomycetes assist our understanding of the cell biology of their simpler cousins?

Our analysis was aided by tabulating reciprocal blastp best hits of the translated products of each *S. coelicolor* gene with those of more than 100 actinobacterial genomes (http://streptomyces.org.uk/actinoblast/). We further analysed these tabulations using different approaches to identify proteins widespread among actinobacteria, but absent from other bacteria (as represented by *E. coli* and *B. subtilis*), in an extension of the work of Gao *et al*. (2006) and Gao & Gupta (2012). These approaches, which we do not describe in detail, included listing proteins in order of their frequency of representation in all actinobacteria analysed and analysing proteins present in both *S. coelicolor* and *Micrococcus luteus*, two morphologically and phylogenetically distinct organisms. Proteins of interest were further investigated using the NCBI Conserved Domain Database, which in several cases proved illuminating in relation to possible function. Throughout the article, the SCO identifiers used in the *S coelicolor* genome are used to designate genes and their protein products interchangeably. The rich genome sequence database used in this survey has caused us to modify some of the conclusions of an earlier exploration of this theme (Chater & Chandra, 2006) and to put forward some new ideas.

## Taxonomy and phylogeny of actinobacteria, in relation to developmental complexity

The taxonomy of actinobacteria has been through several phases. Initially, the phylum consisted of mycelial bacteria termed actinomycetes. Genera were named in accordance with their different modes of sporulation (e.g. *Micromonospora*, *Streptosporangium*, etc.). Subsequently, the use of chemotaxonomy and numerical taxonomy led to the inclusion of some nonmycelial organisms in the phylum. Eventually, the sequencing of 16S ribosomal RNA began to provide a clearer phylogenetic basis for the taxonomy, and further genera of simple bacteria, such as *Bifidobacterium*, were shown to be related to the *Actinomycetales*, leading to the recognition of a more inclusive phylum, *Actinobacteria*. Using such a 16S RNA-based scheme, Zhi *et al*. (2009) divided *Actinobacteria* into five orders, one of which, the *Actinomycetales,* contained the great majority of families, the other four orders being made up of very few families: *Rubrobacterales*, including *Rubrobacter* and *Conexibacter* as genera with sequenced representatives; *Acidimicrobiales,* comprising only *Acidimicrobium*; *Bifidobacteriales*, including the genera *Bifidobacterium* and *Gardnerella*; and *Coriobacteriales*, including sequenced representatives in the genera *Coriobacterium*, *Atopobium*, *Cryptobacterium*, *Eggerthella*, *Olsenella* and *Slackia*. To maximise the ease of relating this article to the existing literature, we are using this taxonomic scheme. However, in the last few years, genome-level information has been employed in various ways to increase the resolution of actinobacterial phylogeny. Alam *et al*. (2010) combined several approaches, including gene order, to arrive at a well-resolved phylogeny, the main limitation of which was the lack of genome sequences representing the deepest branches. A key element in their analysis was the use of catenated sequences of 155 conserved proteins. A catenated set of 21 conserved protein sequences was used by Penn & Jensen (2012) to generate a tree from 186 actinobacterial genome sequences, while Gao & Gupta (2012) used 35 catenated conserved proteins to generate a very well-resolved tree from 98 actinobacterial genomes chosen to give comprehensive coverage of the phylum. Gao & Gupta (2012) went on to show that the distribution of taxon-specific signature indels (small insertions or deletions) and signature proteins fully supported the branch order of their tree, which we have therefore taken as the scaffolding for the rest of this article, but without adopting their revised taxonomic scheme (because it can be confusing for nonspecialists in relation to the pre-existing literature). It was pointed out by Gao & Gupta (2012) that the *Coriobacteriales*, previously included in *Actinobacteria,* lacked all the actinobacterial signature proteins and indels and should therefore be excluded from the phylum (a suggestion re-examined by Gupta *et al*., 2013). Likewise, we found that all the actinobacteria-specific genes that we discuss in this article were absent from *Coriobacteriales*. The term *Actinobacteria* is therefore taken to exclude *Coriobacteriales* throughout this article, although they are included in the reciprocal blastp best-hit analyses shown in some of the figures.

**Fig. 2:**
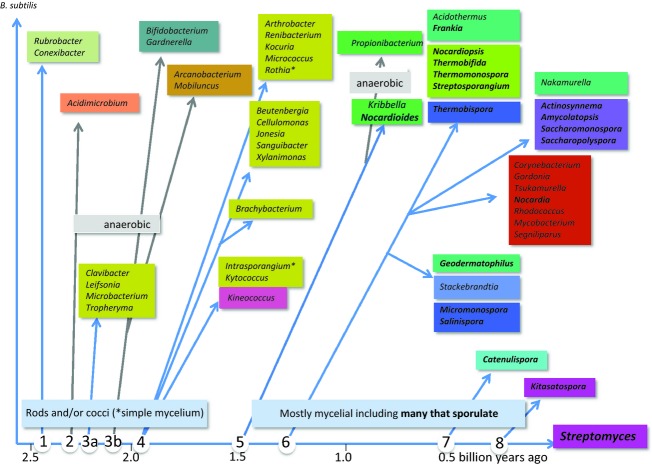
The evolutionary path leading to *Streptomyces*. The diagram was derived from the phylogenetic tree in Fig. 2 of Gao & Gupta (2012), and the boxes correspond in colour to those used in Fig 3. Nodes 1 to 9 are reference points for the main text. Arrow lengths are not proportional to phylogenetic difference. *Micrococcales* genera showing a simple mycelium are indicated by asterisks, and sporulating mycelial genera are given in bold type. The approximate evolutionary timescale is based on Battistuzzi *et al*. (2004).

Because of our underlying emphasis on *Streptomyces,* we needed to re-present this phylogeny from the perspective of *Streptomyces*. To do this, we reorganised the tree of Gao & Gupta (2012) to show clearly the nodes at which various taxa shared a last common ancestor with *Streptomyces*, and aligned it with an estimated timescale derived from Battistuzzi *et al*. (2004) (Fig. 2). A complication of this scheme is the division of one of the *Actinomycetales* suborders, *Micrococcineae*, into three suborders, one of which has a more ancient origin than *Bifidobacteriales* (Gao & Gupta, 2012) and is indicated in Fig. 2 by node 3A. For the purposes of this article, we consider that node 4 of Fig. 2 represents the origin of *Actinomycetales* (i.e. actinomycetes).

It can be seen in Fig. 2 that the actinobacteria originating from nodes 1 to 3B on the path to *Streptomyces* all show variations on a simple rod/coccus morphology and do not sporulate. These organisms include most of the obligately anaerobic genera, consistent with the earliest actinobacteria having preceded the major oxygenation of the atmosphere, around 2.3 Gya (Battistuzzi *et al*., 2004). The earliest branch of the order *Actinomycetales* (node 4) also leads almost exclusively to rod/coccus organisms (suborders *Micrococcineae* and *Kineosporineae*), with (rudimentary) mycelial growth being found only in *Rothia* and *Intrasporangium*. Extensive mycelium formation and sporulation occur in organisms originating at or after node 5, although some genera such as *Corynebacterium* and *Mycobacterium* arising from a later node do not show obvious developmental complexity (readers interested in mycobacterial dormancy or the controversial suggestion that mycobacteria can sporulate are referred to Gengenbacher & Kaufmann, 2012; Lamont *et al*., 2012). The simplest explanation for this discontinuity is that extensive and obligatory mycelial growth arose once and was closely associated with the evolution of sporulation, but such developmental complexity was lost from some lines later in evolution. We show later that loss of complexity is associated with the loss of several developmental regulatory genes.

## The *Streptomyces* sporulation regulatory cascade is built on ancient roots

**Fig. 3:**
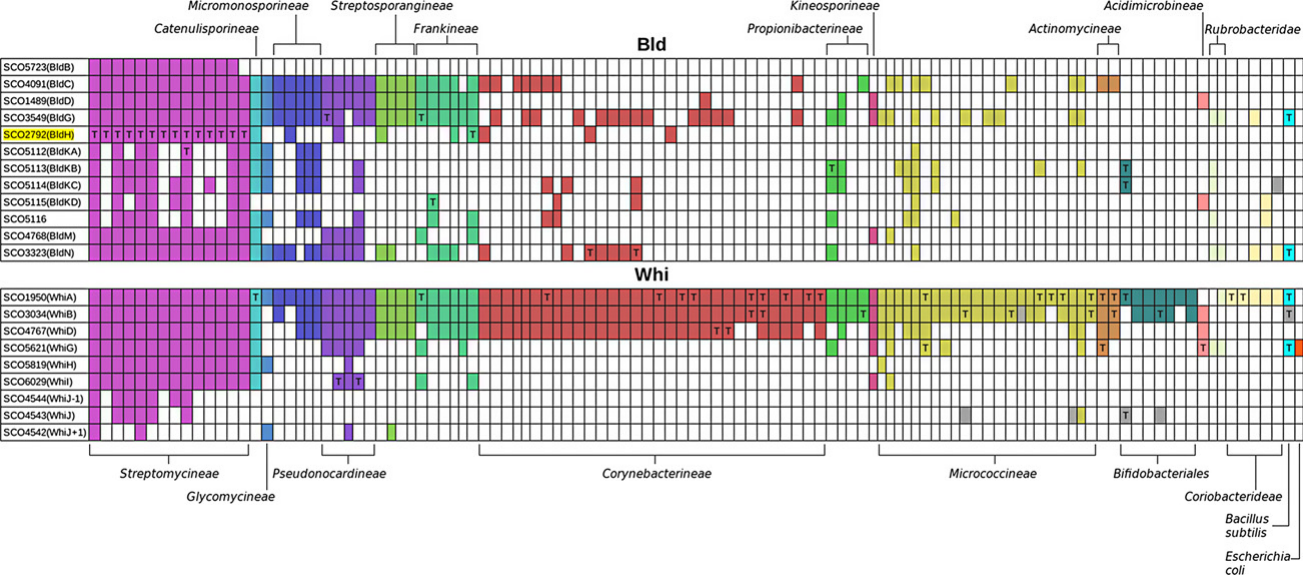
Distribution of probable orthologues of Bld and Whi proteins of *Streptomyces coelicolor* encoded in more than 100 actinobacterial genomes, as detected by reciprocal blastp best hits. Each column represents one genome, and the genomes are grouped and coloured to indicate subgroup relationships (e.g. *Corynebacterineae* columns, including *Mycobacterium*, *Nocardia* and *Corynebacterium*, were coloured Indian red). Grey boxes indicate reciprocal hits falling below the minimal criteria adopted for orthology. White boxes indicate the absence of a reciprocal hit. The yellow highlighted *SCO* genes contain a TTA codon, and the presence of TTA codons in apparent orthologues is indicated by a T in the coloured box. A similar display of reciprocal blastp analysis of the entire *S. coelicolor* genome against the 111 genomes, with links to StrepDB, is available at http://streptomyces.org.uk/actinoblast/. The tables at that site allow clicking onto any coloured box to show the gene identifier together with minimal annotation, as well as information about the length of the overlap and the percentage identity. The sources of genomes are listed in Table 1 of Gao & Gupta (2012). Organisms were as follows (in order across the tabulation). Magenta: *Streptomycineae*, *S. lividans* TK24, *S. viridochromogenes* DSM 40736, *S. scabiei* 87.22, *S. sviceus* ATCC 29083, *S. avermitilis* MA-4680, *S. griseoflavus* Tu4000, *S. venezuelae* ATCC 10712, *S. griseus* subsp. *griseus* NBRC 13350, *S. hygroscopicus* ATCC 53653, *S. pristinaespiralis* ATCC 25486, *S. roseosporus* NRRL 15998, *S. albus* G J1074, *S. clavuligerus* ATCC 27064, *Kitasatospora setae* KM-6054. Turquoise: *Catenulispora acidiphila* DSM 44928. Light blue: *Stackebrandtia nassauensis* DSM 44728. Dark blue: *Salinispora*, *S. tropica* CNB-440, *S. arenicola* CNS-205; *Micromonospora*, *M*. sp. L5, *M*. sp. ATCC39149, *M. aurantiaca* ATCC 27029. Purple: *Saccharomonospora viridis* DSM 43017; *Saccharopolyspora erythraea* NRRL 2338; *Amycolatopsis mediterranei* U32; *Actinosynnema mirum* DSM 43827; *Thermobispora bispora* DSM 43833. Yellow green: *Streptosporangium roseum* DSM 43021; *Thermomonospora curvata* DSM 43183; *Thermobifida fusca* YX; *Nocardiopsis dassonvillei* subsp. *dassonvillei* DSM 43111. Blue green: *Acidothermus cellulolyticus* 11B; *Frankia*, *F*. sp. EAN1pec, *F*. sp. CcI3, *F. alni* ACN14a; *Geodermatophilus obscurus* DSM 43160; *Nakamurella multipartita* DSM 44233. Rust red: *Gordonia bronchialis* DSM 43247; *Nocardia farcinica* IFM 10152; *Segniliparus rotundus* DSM 44985; *Tsukamurella paurometabola* DSM 20162; *Rhodococcus*, *R. opacus* B4, *R. jostii* RHA1, *R. erythropolis* PR4, *R. equi* 103S; *Mycobacterium*, *M. vanbaalenii* PYR-1, *M. ulcerans* Agy99, *M*. sp. Spyr1, *M*. sp. MCS, *M*. sp. KMS, *M*. sp. JLS, *M. smegmatis* str. MC^2^ 155, *M. marinum* M, *M. leprae* Br4923, *M. gilvum* PYR-GCK, *M. abscessus* ATCC 19977, *M. avium* subsp. *paratuberculosis* K-10, *M. avium* 104, *M. tuberculosis* H37Rv, *M. bovis* AF2122/97; *Corynebacterium*, *C. urealyticum* DSM 7109, *C. pseudotuberculosis* FRC41, *C. kroppenstedtii* DSM 44385, *C. jeikeium* K411, *C. glutamicum* ATCC 13032 2, *C. efficiens* YS-314, *C. diphtheriae* NCTC 13129, *C. aurimucosum* ATCC 700975. Bright green: *Nocardioides* sp. JS614; *Kribbella flavida* DSM 17836; *Propionibacterium*, *P. freudenreichii* subsp. *shermanii* CIRM-BIA1, *P. acnes* KPA171202. Plum: *Kineococcus radiotolerans* SRS30216. Olive yellow: *Beutenbergia cavernae* DSM 12333; *Cellulomonas flavigena* DSM 20109; *Brachybacterium faecium* DSM 4810; *Kytococcus sedentarius* DSM 20547; *Intrasporangium calvum* DSM 43043; *Jonesia denitrificans* DSM 20603; *Clavibacter michiganensis* subsp. *michiganensis* NCPPB 382; *Leifsonia xyli* subsp. *xyli* str. CTCB07; *Microbacterium testaceum* StLB037; *Arthrobacter*, *A*. sp. FB24, *A. phenanthrenivorans* Sphe3, *A. chlorophenolicus* A6, *A. aurescens* TC1, *A. arilaitensis* Re117; *Kocuria rhizophila* DC2201; *Micrococcus luteus* NCTC 2665; *Renibacterium salmoninarum* ATCC 33209; Rothias, *R. mucilaginosa* DY-18, *R. dentocariosa* ATCC 17931; *Xylanimonas cellulosilytica* DSM 15894; *Sanguibacter keddieii* DSM 10542; *Tropheryma whipplei* str. Twist. Brown: *Mobiluncus curtisii* ATCC 43063; *Arcanobacterium haemolyticum* DSM 20595. Cyan: *Gardnerella vaginalis* ATCC 14019; *Bifidobacterium, B. longum* NCC2705, *B. longum* DJO10A, *B. dentium* Bd1, *B. bifidum* PRL2010, *B. animalis* subsp. *lactis* Bl-04, *B. adolescentis* ATCC 15703. Pink: *Acidimicrobium ferrooxidans* DSM10331. Pale grey green: *Conexibacter woesii* DSM14684; *Rubrobacter xylanophilus* DSM9941. Beige: *Atopobium parvulum* DSM 20469; *Cryptobacterium curtum* DSM 15641; *Eggerthella lenta* DSM 2243; *Olsenella uli* DSM 7084; *Slackia heliotrinireducens* DSM 20476.

Focused genetic studies of model streptomycetes have revealed several tens of key developmental genes (Flärdh & Buttner, 2009; Chater, 2011; McCormick & Flärdh, 2012). Mutations in some of these genes result in the loss of aerial mycelium formation, at least under most culture conditions (Merrick, 1976; Champness, 1988). Because of the bald appearance of the colonies, such genes are mostly designated *bld*. Another major phenotypic class of developmental mutants – those that form aerial hyphae but do not sporulate efficiently – identified the *whi* genes, so-called because the mutants fail to accumulate spore pigment in their aerial mycelium, which remains white on prolonged incubation (Hopwood *et al*., 1970; Chater, 1972). Here, we evaluate the phylogenetic distribution of many of these genes and interpret the results in terms of the evolution and mechanisms of *Streptomyces* development (Fig. 3: the legend to Fig. 3 includes information about the methods used to generate the data and how to access the full tables). Unless stated otherwise, orthologues of these genes are absent from *B. subtilis* and *E. coli*, so they may well be confined to actinobacteria. It appears from this that the key developmental regulatory roots of *Streptomyces* sporulation described in this section lie in some of the Whi proteins, while actions of the Bld proteins (also mostly regulatory) have come to be overlaid on the initiation of the *whi* gene cascade. These genes are discussed in the inferred order of their appearance during the c. 2.7 G years since the emergence of the first actinobacteria (Battistuzzi *et al*., 2004). We also identify some potentially interesting, but sometimes little-studied, genes whose patterns of occurrence across the actinobacteria are congruent with those of certain well-known developmental genes, and speculate on the significance of this congruence.

### WhiG, an orthologue of an ancient sigma factor, regulates more recently acquired regulatory genes specific to aerial sporulation

Considering likely orthologues of all the *bld* and *whi* genes studied, none is more widespread across the bacterial kingdom than *whiG*. WhiG protein is a sigma factor critically involved in the decision of aerial hyphae to sporulate, and in its absence, colonies develop long, thin aerial hyphae and entirely fail to sporulate (Chater, 1972). It is orthologous with the extensively studied FliA of *E. coli* and SigD of *B. subtilis*, which are involved in regulating genes important for motility and chemotaxis, adhesion and invasion, some aspect(s) of cell wall remodelling and cyclic di-AMP hydrolysis (Helmann, 1991; Claret *et al*., 2007; Luo & Helmann, 2012). It is possible to envisage connections between these functions and *Streptomyces* sporulation, as they are mostly associated with the transition from growth as a biofilm to dispersal as planktonic single cells. However, FliA in *E. coli* and SigD in *B. subtilis* are both regulated by an antisigma factor, FlgM, that has the extraordinary property of being exported via the flagellar basal body during flagellum assembly. This is clearly not feasible for nonmotile streptomycetes, so it is not surprising that no homologue of this antisigma factor has been found in streptomycetes.

WhiG orthologues are widely but intermittently present in diverse actinobacteria, including some that are morphologically simple (Fig. 3). In most cases, these simpler organisms have been recorded as motile, the exceptions being *Acidithermus* and *Rubrobacter* (but *Acidithermus* does have a set of flagellar genes: Barabote *et al*., 2009). The only node 6-branch organism possessing WhiG, *Nocardioides*, is the only mycelial, sporulating organism known in this branch, and it also has a set of flagellar genes (Barabote *et al*., 2009). Even if motility functions are regulated by WhiG orthologues in these actinobacteria, no FlgM-like protein is encoded in any of their genomes. The *whiG*-like genes all show some local synteny, part of which is even retained in *B. subtilis,* so *whiG* seems to have been lost independently from several actinobacterial lines, rather than having been absent from the last common ancestor and then reacquired later in actinobacterial evolution as we previously suggested (Chater & Chandra, 2006).

RNA polymerase containing WhiG sigma directly activates two regulatory genes involved in slightly later stages in sporulation (*whiH*, Ryding *et al*., 1998; *whiI*, Ainsa *et al*., 1999). WhiI protein resembles response regulators, many of which are part of two-component systems in which activity of the response regulator is determined by its phosphorylation by a partner sensor kinase. WhiI, however, does not have a known partner kinase, being one of 13 ‘orphan’ response regulators present in *S. coelicolor* (Hutchings, 2007), and lacks key residues normally required for phosphorylation (Tian *et al*., 2007). It occurs almost exclusively in developmentally complex WhiG-containing actinomycetes and is absent from WhiG-containing, morphologically simple, motile actinobacteria; but both WhiG and WhiI are absent from many mycelial actinomycetes whose sporulation does not involve the formation of chains of spores on long aerial hyphae (*Frankia*, *Micromonospora*, *Salinispora*, *Thermobispora*, *Nocardiopsis*, *Thermobifida*, *Streptosporangium* and *Thermomonospora*). The other WhiG target regulatory gene, *whiH*, encodes an autoregulating GntR-like protein (Ryding *et al*., 1998; Persson *et al*., 2013) confined to streptomycetes and their closest relatives (*Catenulispora* and *Kitasatospora*).

In summary, the WhiG-dependent part of the *Streptomyces* sporulation regulatory cascade (as known until recently, see below) appears to have evolved in a stepwise manner, in which an early role for WhiG may have been to facilitate planktonic dispersal from biofilms (but there is still no analysis of roles for WhiG in motility and chemotaxis of motile simple actinobacteria). This made it potentially appropriate for activating the analogous process of sporulation of mycelial mats. The subsequent acquisition (and WhiG dependence) of WhiI and WhiH may have permitted increased provision of components needed in large amounts for sporulation septation and spore maturation, as in WhiI-dependent upregulation of genes needed for phosphoinositides for membrane synthesis (Tian *et al*., 2007; Zhang *et al*., 2012) and an apparently WhiH-stimulated increase in the supply of FtsZ for sporulation septation (Flärdh *et al*., 1999, 2000).

### *whiA*, part of a syntenous cluster of genes conserved across Gram-positive bacteria

Like WhiG (but no other sporulation regulator of *S. coelicolor*), WhiA orthologues are not confined to actinobacteria: one is present in most Gram-positive bacteria, including all actinobacteria except *Acidimicrobium ferrooxidans*. The structure and molecular function of WhiA have only been fruitfully studied recently. One of its two domains is an evolutionary relative of homing endonucleases, but lacks catalytic residues, and the other resembles the C-terminal domain of major sigma factors, which interacts with the -35 region of promoters (Knizewski & Ginalski, 2007; Kaiser *et al*., 2009). WhiA showed *in vitro* DNA binding to its own promoter and to a sporulation-activated promoter of the *parAB* operon (Kaiser & Stoddard, 2011), both of which are also WhiA-dependent *in vivo* (Jakimowicz *et al*., 2006). The *whiA* sporulation-specific promoter could be transcribed *in vitro* by WhiG-containing RNA polymerase (Kaiser & Stoddard, 2011), in contradiction of an earlier result (Ainsa *et al*., 2000). WhiA exerted a modest inhibitory effect on this transcription and showed some evidence of direct interaction with WhiG in a pull-down experiment involving the two purified proteins (Kaiser & Stoddard, 2011). These experiments, although not conclusive, provide the first suggestion of direct interplay between the WhiG- and WhiA-dependent parts of the sporulation regulatory cascade, previously thought to be separate (Chater, 1998; Flärdh *et al*., 1999).

*whiA* and the upstream three genes form a cluster that is highly conserved in actinobacteria and even in *B. subtilis*. This putative operon is probably responsible for a low level of *whiA* (*SCO1950*) expression during growth (Ainsa *et al*., 2000). The three upstream genes encode apparently unrelated deduced functions: the UvrC excinuclease (SCO1953); a NTPase that inactivates an sRNA (GlmZ) that regulates glucosamine-6-phosphate (GlcN6P) synthase production in *E. coli* (NCBI conserved domain PRK05416; SCO1952); and a protein of unknown function (SCO1951) that is related to an enzyme of cytochrome F420 biosynthesis, LPPG:Fo 2-phospho-L-lactate transferase (pfam01933). There is also conspicuous synteny on the other side of *whiA* in actinobacteria (but this does not extend to *B. subtilis*): three genes for steps in glycolysis/gluconeogenesis, glyceraldehyde-3-phosphate dehydrogenase, phosphoglycerate kinase and triose phosphate isomerase, are always found next to *whiA* (or separated from it by one or two genes in some streptomycetes), along with *secG*, encoding part of the protein secretion system. If the notion of ‘guilt by association’ is applied to *whiA*, we may guess that it operates in the context of a physiological transition resulting from nutritional limitation, such that assimilated nutrients are redirected via gluconeogenesis to generate glucose-6-phosphate, which may then be converted into N-acetyl glucosamine for cell wall synthesis during aerial growth (perhaps also feeding into mycothiol biosynthesis, see below). This model does not account for *all* the conserved genetic linkage of *whiA*, but it is consistent with the apparent inability of aerial hyphae of *whiA* mutants of streptomycetes to stop growing and switch to sporulation (Chater, 1972).

### WhiB and its paralogues: ancient actinobacterial nitric oxide-binding proteins

A phenotype identical to that of *whiA* mutants results from mutations in *whiB* (*SCO3034*), which encodes one of the actinobacterial signature proteins (Table 1A, Fig. 3). Mutation of the *whiB* orthologue (*whmD*) of *Mycobacterium smegmatis* indicated a likely role in cell division that could represent its core activity (Gomez & Bishai, 2000). There are strong two-way transcriptional influences (not necessarily direct) between *whiA* and *whiB* (Jakimowicz *et al*., 2006), but little is known about other possible WhiB targets.

**Fig. 4:**
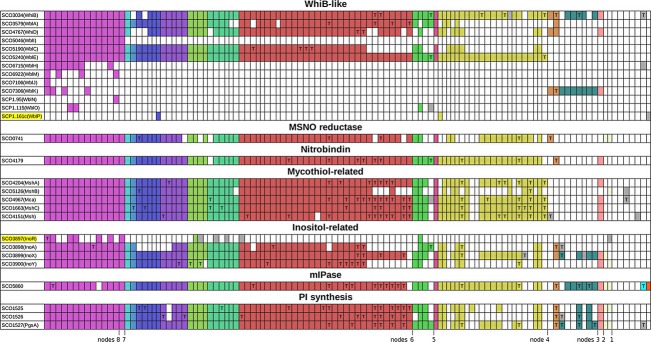
Distribution of WhiB-like (Wbl) proteins compared with proteins related to nitric oxide and mycothiol metabolism. The numbered nodes refer to Fig. 2. See Fig. 3 legend and text for further details.

WhiB is the exemplar of a paralogous family of small proteins (Wbl for WhiB-like: Soliveri *et al*., 1993, 2000; Fowler-Goldsworthy *et al*., 2011) that all possess an oxygen-sensitive [4Fe, 4S] cluster coordinated by four conserved cysteinyl residues (Jakimowicz *et al*., [Bibr b115][Bibr b116]; den Hengst & Buttner, 2008; Alam *et al*., 2009; Saini *et al*., 2011, 2012). Orthologues of four other Wbl proteins (WblA, WblC, WhiD and WblE) occur in most actinomycetes (Figs 3 and 4), even though WblA and WhiD have developmental roles in *S. coelicolor*: WblA plays a key part in the transition of aerial hyphal initial branches to a sporulation-directed fate (*wblA* mutants have thin aerial hyphae often embedded in an extracellular matrix, with only occasional spore chains: Fowler-Goldsworthy *et al*., 2011); and mutants lacking WhiD have defects at a later stage, having thin-walled spores and uncontrolled sporulation septation (McVittie, 1974; Molle *et al*., 2000). Limited information is available about the roles of these two proteins in simpler actinobacteria: in *Corynebacterium glutamicum,* the WblA orthologue WhcA negatively influences the oxidative stress response (Choi *et al*., 2009); and the WhiD orthologue of *Mycobacterium tuberculosis* (WhiB3) is required for virulence (Saini *et al*., 2011). Some nonactinomycete actinobacteria also contain Wbl proteins, notably *Bifidobacteriales,* which have orthologues of two *S. coelicolor* Wbls, WhiB and WblK, respectively, termed WhiB2 and WblE in a recent survey (Averina *et al*., 2012). Using WhiB as a probe in one-way blastp searches of all the translated actinobacterial genomes, paralogues were sometimes very abundant: in an extreme case, *Rhodococcus jostii* possessed 30 *wbl* genes, all but five of them being almost specific to this organism or genus. The frequent finding of *wbl* genes in actinophages (e.g. Dedrick *et al*., 2013) and plasmids (e.g. SCP1; Fig. 4 and Bentley *et al*., 2004) makes it plausible that the large family of Wbl paralogues evolved in these elements, which would also serve as agents for their genome-specific lateral acquisition by diverse actinobacteria (Saini *et al*., 2011).

Soliveri *et al*. (2000) suggested that WhiB and other Wbl proteins might interact with the major antioxidant thiol mycothiol (MSH), which is widespread among, and apparently confined to, actinobacteria (Fahey, 2012). Genomic searches confirmed that the MSH pathway is present in most *Actinomycetales*, but it is absent from nonactinomycete actinobacteria apart from *Acidimicrobium ferrooxidans*, even though *wbl* genes are present in many of these (Fig. 4). Thus, Wbl proteins can fulfil at least some function(s) in the absence of MSH. Nevertheless, another Wbl protein of *M. tuberculosis*, WhiB7 (=WblC), which is important in a global response to various antibiotics and other inhibitors and is widespread among actinobacteria, was found to control, directly or indirectly, the concentration of mycothiol (MSH+MSSM; Morris *et al*., 2005; Burian *et al*., 2012), and mycothiol-deficient mutants of *Mycobacterium smegmatis* (Rawat *et al*., 2002), *Rhodococcus jostii* (Dosanjh *et al*., 2008) and *Corynebacterium glutamicum* (Liu *et al*., 2013b) showed pleiotropic sensitivity to antibiotics similar to that of *whiB7/wblC* mutants of *M. tuberculosis* and *Streptomyces lividans* (Morris *et al*., 2005).

Several Wbl proteins have been shown to have specific DNA-binding activity (Rybniker *et al*., 2010; Smith *et al*., 2010; Stapleton *et al*., 2012). This can be enhanced by rapid, high-affinity interaction of the [4Fe, 4S] clusters with NO (Singh *et al*., 2007; Smith *et al*., 2010; Crack *et al*., 2011, 2013; Stapleton *et al*., 2012). Interestingly, we found two likely NO-related genes with a phylogenetic distribution similar (although not identical) to that of Wbl proteins (Fig. 4). One of these genes, *SCO0741*, encodes an orthologue of a mycobacterial protein that *in vitro* very rapidly reduces an NO conjugate (MSNO) of MSH to MSH sulphonamide, which *in vivo* is processed by *M. smegmatis* to MSSM (oxidised MSH) and nitrate (Vogt *et al*., 2003).

**Fig. 5:**
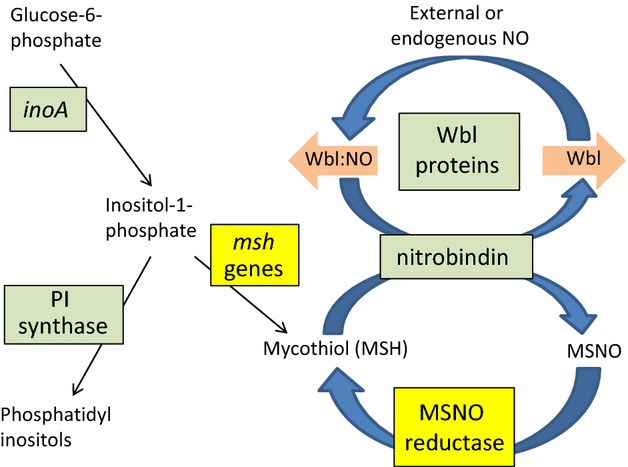
Hypothetical scheme invoking the involvement of nitric oxide, mycothiol and Wbl proteins in major physiological or developmental decisions. It is supposed that early actinobacteria possessed the functions coloured grey green. They made phosphoinositol-containing phospholipids and used Wbl proteins to respond to nitrosative stress (the pink arrows indicate downstream regulatory events of different Wbl states). The putative nitrobindin may have aided the denitrosylation of Wbl:NO proteins. It is further suggested that the subsequent acquisition of mycothiol biosynthetic genes and MSNO reductase greatly increased the efficiency of NO removal and Wbl regeneration.

The second potentially NO-related protein with a very similar distribution, SCO4179, has clearcut similarity to nitrobindins of plants and animals (Bianchetti *et al*., 2010; Bianchetti *et al*., 2011). Nitrobindins are haem-containing proteins that bind NO in the absence of oxygen, and whose major structural features are conserved in the SCO4179 protein and its orthologues in other actinobacteria (Shepard *et al*., 2007; Bianchetti *et al*., 2010, 2011). A potential role for nitrobindin might be to transfer the NO groups of Wbl:NO complexes to MSH (Fig. 5). This would provide a means of recycling Wbl proteins to ensure that any burst of Wbl:NO-dependent transcription would be switched off once the Wbl-dependent gene expression cascade had been set in motion (Fig. 5). MSNO might in turn be denitrosylated by MSNO reductase. The MSSM formed in this process would then be reduced to MSH either by mycothiol reductase, which is present in most actinomycetes although apparently not in streptomycetes, or by some other, possibly less specific, thiol reductase. The MSNO reductase gene is immediately upstream of a gene (*SCO0740*) encoding a protein with homology to hydroxyacylglutathione hydrolases (Rawat & Av-Gay, 2007). This pairing is seen in almost all the actinobacteria that possess the MSNO reductase gene, with the genes usually overlapping by one nucleotide, indicating likely co-transcription and translational coupling. As glutathione is not present in most actinobacteria, an activity on a mycothiol derivative may be the real function of SCO0740 – perhaps in association with MSNO reductase.

A further hint of a Wbl–NO connection has been found: in *Corynebacterium glutamicum,* the Wbl protein WhcA appears to interact with a protein showing very high similarity to nitronate monooxygenase (Park *et al*., 2011), an FMN-dependent fungal and bacterial enzyme that generates nitrite from alkyl nitronates (Gadda & Francis, 2010) and is found in nearly all actinobacteria (the *S. coelicolor* equivalent of this protein is SCO2553).

If MSNO was significant for early actinobacteria that emerged *before* the evolution of complex eukaryotes that produce NO as a defence and signalling molecule, NO may be an endogenous signal molecule in actinobacteria, which in *Streptomyces* fulfils roles in development (and in any other general physiological changes influenced by Wbl proteins). How might NO be generated, as the great majority of actinobacteria do not possess an obvious nitric oxide synthase? In plants, nitrate reductase has been implicated as a generator of endogenous NO that brings about the closure of stomata (Desikan *et al*., 2002), and nitrate and nitrite reductases generate NO in bacteria (Corker & Poole, 2003; Vine *et al*., 2011). Like the binding of NO by nitrobindin (Bianchetti *et al*., 2010), these reactions are anoxic. This may explain why the WhiB7 (=WblC)-dependent response of *M. tuberculosis* to antibiotics was surprisingly stimulated by reducing conditions (added dithiothreitol), but not by oxidative stress induced by the thiol oxidant diamide (Burian *et al*., 2012). It is interesting to note that in surveys of the thiol-oxidative stress responses mediated by the SigR system, only one of the genes discussed in this section (*mshA*, determining a step in MSH biosynthesis) was part of the SigR regulon (Paget *et al*., 2001; Kim *et al*., 2012). This is consistent with the idea of a partial separation of Wbl–NO–MSH physiology from responses to external oxidative stress (but does not exclude them from having some involvement).

The acquisition of MSH biosynthesis early in actinobacterial evolution seems to have been preceded by the means to generate an immediate precursor for MSH, myoinositol-1-phosphate (mIP), which is also absent from other bacteria. The relevant gene (*SCO3899*, *inoA*), is present in at least one nonactinomycete, *Arcanobacterium haemolyticum*, and is present in many actinomycetes, although surprisingly absent from corynebacteria and many *Micrococcales*. The source of inositol for MSH biosynthesis in these organisms is not known. When present, *inoA* is generally adjacent to its regulatory gene (*inoR*; Zhang *et al*., 2012). A similar distribution across actinobacteria was found for the biosynthetic genes of other inositol derivatives such as phosphoinositides (a three-gene cluster comprising *SCO1527*, putatively encoding phosphoinositide synthase, and *SCO1525* and *SCO1526*, likely determinants of the further modification of phosphoinositides: Zhang *et al*., 2012; Fig. 4). From this, it seems likely that an early actinobacterial organism already possessed the ability to make phosphoinositides from glucose-6-phosphate and that the later acquisition of MSH biosynthesis, close to the time of emergence of the first actinomycetes, was made possible by the availability of the MSH precursor mIP (Fig. 5).

Finally in this section, we draw attention to genes for two further signature proteins listed in Table 1B: *SCO1664* and SCO*4205* are invariably closely linked to MSH biosynthetic genes (*mshC*, *SCO1663*, and *mshA*, *SCO4204*), so it is possible that their functions may also be implicated in the network proposed in Fig. 5.

### Evolution of the developmental roles of two ancient genes, *bldC* and *bldD*

In *B. subtilis*, sporulation is an extreme response to nutrient limitation usually taken only when all other solutions fail (Narula *et al*., 2012). Likewise, in streptomycetes, it seems that many (but not all) of the *bld* gene products, which are nearly all regulatory, feed in information relevant to this drastic decision and ensure that the *whi* gene cascade operates only under fully appropriate circumstances.

The most ubiquitous *bld* genes are *bldC*, encoding an apparently single-domain small protein with a helix-turn-helix of the MerR type (Hunt *et al*., 2005), and *bldD*, which encodes a protein distantly related to SinR, a transition state regulator of *B. subtilis* (Elliot *et al*., 1998). Orthologues of both are found in most of the morphologically complex, large-genome actinomycetes. Simpler organisms (relatively anciently diverged from the *Streptomyces* line) seldom have both, and often (as in the case of anaerobic actinobacteria) have neither (Fig. 3). *bldD* orthologues always show high conservation and local synteny, but *bldC* orthologues are somewhat less highly conserved and are located among less extensively conserved genes, although some evidence of *bldC* synteny could often be detected (Chater & Chandra, 2006) (except where the BldC reciprocal blastp best hits were to proteins showing well under 50% identity – such cases may well be laterally acquired paralogues). Importantly, there are convincing orthologues of *bldC* in *Rubrobacteriales* and *bldD* in *Acidimicrobium*. Thus, both genes were present in very early actinobacteria, but each gene has been lost many times in the later evolution of the phylum. These losses may conceivably have contributed to the evolution of branches such as *Micrococcineae* (Node 4 of Fig. 2) and *Corynebacterineae* (a sub-branch from node 6).

The BldD regulon has been subjected to detailed analysis by immunoprecipitation of *in vivo* BldD–DNA complexes, which showed that BldD directly targets about 147 transcription units in vegetative, liquid-grown *S. coelicolor* (den Hengst *et al*., 2010). These include 42 regulatory genes, several of which are developmental (*bldA, bldC, bldD, bldH, bldM, bldN, whiB, whiG*). These are all repressed by BldD. Based on a consensus sequence derived from these ChIP-chip data, BldD recognition sequences were found upstream of many of the same genes not only in other streptomycetes, but also in other sporulating actinobacteria (den Hengst *et al*., 2010). Such species included *Saccharopolyspora erythraea*, an organism in which a constructed *bldD* mutant had a bald colony phenotype (Chng *et al*., 2008). Thus, BldD orthologues appear to coordinate development in diverse sporulating actinomycetes, perhaps preventing the expression of genes for morphological differentiation and antibiotic production during vegetative growth and connecting the regulons of other regulators of these processes (den Hengst *et al*., 2010). BldD orthologues in simpler actinomycetes might well have roles both during growth, to repress functions associated with entry into stationary phase, and in stationary phase, in coordinating the expression of different stationary phase regulatory genes.

Despite the extensive characterisation of BldD and its regulon, it is not understood why, if BldD represses developmental functions, *bldD* mutants are bald rather than hypersporulating (but see the paragraphs on BldN below); and there is no information about any signals that BldD might respond to (an initial search for possible proteins interacting with BldD was reported to have had negative results: den Hengst *et al*., 2010). It has been suggested that an interaction of BldD with another sporulation regulatory protein, BldB, could determine the rate of turnover of BldD (McCormick & Flärdh, 2012); but BldB is confined to streptomycetes, so it could not fulfil such a role in other complex actinomycetes such as *Sac. erythraea* (Fig. 3).

#### Evolution of the *BldD* regulon

Some BldD-regulated *bld* genes of *S. coelicolor* belong to classes of genes that are widespread and often represented by multiple paralogues in any one genome. For such genes, it can be difficult to be confident that reciprocal blastp hits between genomes are meaningful, particularly when the extent of amino acid identity falls well below levels that are typically seen for conserved housekeeping genes. For example, the general kind of anti-anti-sigma factor to which the BldG protein belongs is almost universally found among both Gram-positive firmicutes and actinobacteria; so the presence in some actinobacteria of BldG reciprocal best hits with identities only in the 20–40% range is relatively uninformative (in fact, such low-scoring hits did not show the local synteny seen with those having higher identities). Evidently, anti-anti-sigmas (and corresponding antisigmas) of this general class were present in the ur-actinobacterium, giving rise to the possibility of subtle control of sigma factor activity by signals that might include morphological checkpoints (as in the case of the *spoIIAA*/*spoIIAB* genes of *B. subtilis*; Piggot & Hilbert, 2004) or stress (as in the case of *sigB* of *B. subtilis*; Price, 2000). Indeed, BldG influences the activity of the stress-responsive sigma factor SigH in *S. coelicolor* (Sevcikova *et al*., 2010; Takano *et al*., 2011), and the anti-anti-sigma/antisigma/sigma interactions of this general type have considerable potential for promiscuity in *Streptomyces* (Kim *et al*., [Bibr b127][Bibr b128]; Sevcikova *et al*., 2010; Takano *et al*., 2011).

The problem of recognising orthologues among large families of paralogues is less severe with the phylogenetically distinct ECF class of sigmas and their antisigma partners, which are more diverse than the class regulated through BldG-like cascades, and usually show high partner specificity (Staron *et al*., 2009). *bldN*, a direct target of BldD, encodes one of about 50 *S. coelicolor* ECF sigma factors (Bibb *et al*., 2000; den Hengst *et al*., 2010). At least in *S. venezuelae*, BldN is a direct activator of the genes for chaplins (and their associated rodlins): amphipathic proteins that assemble at air–water interfaces and coat incipient aerial hyphae, facilitating their emergence into the air (Bibb *et al*., 2012; see below). This emergence into the air has been suggested as a trigger for the sporulation pathway controlled by the *whi* genes (Claessen *et al*., 2006). Convincing BldN reciprocal hits (at well over 50% identity and with local synteny) were found only among morphologically complex genera of actinomycetes (Fig. 3), suggesting a close connection of *bldN* with the emergence of complexity (reciprocal hits with other actinobacteria were all at well under 40% identity and lacked discernible synteny).

**Fig. 6:**
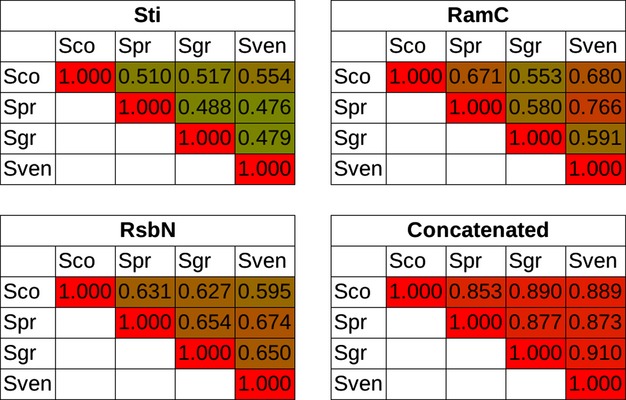
High rates of divergence of three developmentally significant proteins. Pairwise blastp comparisons are shown between three developmental proteins of four *Streptomyces* spp: *S. coelicolor* (Sco), *S. pristinaespiralis* (Spr), *S. griseus* (Sgr) and *S. venezuelae* (Sven). High percentage identity is indicated by the intensity of red, and low identity by the intensity of green. A control table (‘Concatenated’) shows the comparisons for a concatenated set of seven universal proteins (AtpD, DnaA, DnaG, DnaK, GyrB, RecA, RpoB).

It has been demonstrated that, in *S. venezuelae,* an antisigma factor controlling BldN is encoded by the adjacent gene, termed *rsbN* (= *SCO3324* in *S.coelicolor*; Bibb *et al*., 2012). In blastp analysis, a reciprocal best hit to *rsbN* is found next to nearly all *bldN* orthologues in actinomycete genomes; but, strikingly, the RsbN-like proteins are much more divergent than their BldN target or most other families of orthologous proteins of actinobacteria (Fig 6). We speculate that this may imply differences in the signal responsiveness of different RsbN proteins, thereby contributing to the differences between different organisms in the interplay of ecology and development: in other words, they may be potential agents of speciation.

The *rsbN* gene of *S. venezuelae* has its own promoter, which is BldN-dependent, and is also a BldD target (den Hengst *et al*., 2010; Bibb *et al*., 2012). As a *bldD* mutant might therefore be expected to overexpress *rsbN*, the resulting increase in anti-BldN activity might interfere with the expression of BldN-dependent genes and contribute significantly to the bald phenotype of *bldD* mutants.

The most well-studied target of BldN is *bldM*, which encodes an orphan response regulator (Molle & Buttner, 2000). The distribution of convincing reciprocal hits to *bldM* is closely similar to that of *bldN* hits, suggesting that the BldN to BldM regulatory step was established very early in the evolution of actinomycete complexity. The distribution of BldM was even more closely similar to that of orthologues of another developmental orphan response regulator, WhiI (Fig. 3).

### The key developmental regulator AdpA emerged along with complex mycelial growth and is *bldA*-dependent only in *Streptomycineae*

BldD targets also include *adpA*, known as *bldH* in *S. coelicolor* (den Hengst *et al*., 2010). AdpA has been most comprehensively described in *S. griseus*, in which it is the agent of the effects of the hormone-like A-factor (Horinouchi, 2002). It comprises a structurally characterised C-terminal AraC/XylS-like DNA-binding domain (Yao *et al*., 2012) and an N-terminal domain that may sense adenine nucleotides (Wolanski *et al*., 2012; Liu *et al*., 2013a). It plays a central role in the decisions leading to colony differentiation, notably affecting extracellular functions such as protease cascades, extracellular morphogenetic peptides and secondary metabolism (Akanuma *et al*., 2009; Chater *et al*., 2011; Higo *et al*., 2012), but also contributing to the regulation of DnaA-mediated chromosome replication initiation (Wolanski *et al*., 2012). In *S. griseus*, many hundreds of direct targets for AdpA have been defined, and it is suspected that the unusually low DNA-binding specificity of AdpA may permit the ready recruitment of new targets, leading to species-specific differences in AdpA regulons (Higo *et al*., 2012). The phylogenetic distribution of *adpA*-like genes is similar to that of *bldN*-like genes (Fig. 3), but there is little evidence of direct regulatory interplay between the two genes. Possibly, then, AdpA evolved to regulate aspects of developmental physiology complementary to those regulated by BldN (if so, one might anticipate that some cross-checks between the two regulons will eventually be discovered).

The regulation of *adpA* in streptomycetes is remarkably complex (reviewed in detail in Liu *et al*., 2013a). It involves at least three levels of control: transcriptional [autorepression (Kato *et al*., 2005), repression by BldD (den Hengst *et al*., 2010), repression by gamma-butyrolactone-binding proteins (Horinouchi, 2007; Xu *et al*., 2009)]; mRNA processing by RNaseE (Xu *et al*., 2010); and mRNA translation (Nguyen *et al*., 2003; Takano *et al*., 2003). Translational regulation is via a very rare UUA codon in the *adpA* mRNA, falling between the segments encoding the two domains of AdpA. UUA is the only one of the six leucine codons to comprise only A and U residues, so the corresponding TTA codon is comparatively rare in GC-rich genomes – it occurs in only 147 chromosomal genes in *S. coelicolor* (Li *et al*., 2007). UUA codons have a special regulatory role in *Streptomyces*, as indicated by the finding that mutants (*bldA*) in the gene for the UUA-reading tRNA grow well, but fail to form aerial mycelium or some antibiotics (Merrick, 1976; Lawlor *et al*., 1987). *adpA* is the only gene that has a TTA codon in all the streptomycetes analysed (Fig. 3; Table 2; Chater & Chandra, 2008), a feature also found in the *adpA* orthologue in *Kitasatospora setae*. The TTA codon in *adpA* was shown by mutagenesis to be the main (but not entire) cause of the Bld phenotype of *bldA* mutants of *S. coelicolor* (Nguyen *et al*., 2003; Takano *et al*., 2003). A study of *S. griseus* and *S. coelicolor* has shown that the abundance of *bldA* tRNA is important in determining whether AdpA reaches levels sufficient to activate development and, remarkably, that there is a mutual feedforward mechanism in which AdpA activates *bldA* transcription (Higo *et al*., 2011). However, the *adpA*-like genes of other actinomycetes, including *Catenulispora acidiphila* (the closest genome-sequenced relative of *Streptomyces* and *K. setae*), are nearly all TTA-free (in the single exception, *Nakamurella multipartita*, the TTA codon is not located in the interdomain-coding region, but close to the 3′-end of the gene). Thus, *bldA*-*adpA* interplay was apparently established after node 7 (Fig. 2), branching to *Catenulispora*, but before the *Streptomyces* and *Kitasatospora* lines diverged (node 8). Indeed, the broader developmental significance of *bldA* may not extend beyond *Streptomycineae*, as in non-*Streptomycineae* genomes TTA codons do not show the positional bias towards the start of genes that is observed in streptomycetes, and sometimes occur in conserved growth-associated genes (Chater & Chandra, 2008). Interestingly, there is a strong target for BldD binding within *bldA* (den Hengst *et al*., 2010).

**Table 2:** *Streptomyces* genes or gene clusters that frequently contain TTA codons*

*SCO* number of gene *asterisk means TTA codons are absent from the *S. coelicolor* gene	Function	Fraction of TTA-containing orthologues among 14 *Streptomyces* genomes (see note)	*K. setae* orthologue, accepting > 40% identity (TTA present?)
0381*;0382*;*0383*	Enzymatic modifications	6/12; 5/9; 2/10	–
0683*;0684*;0685*	Unknown	2/12; 4/13; 2/13	*67090* (–) *67100 (–) *67110 (–)
*1187*(*celB*);1188* (celS2*)	Cellulose utilisation	6/13; 7/14	– *59340 (–)
*1242*	DNA-binding regulatory protein (WhiJ-like)	9/11	*58780* (–)
1256*	Unknown	6/10	–
*1434*	Secreted AAA ATPase	8/13	*11520* (–)
*1592*	ADPribose pyrophosphatase	8/12	*41440* (–)
*1980*	Possible antisigma factor (AbaA-like)	9/14	–
*2426*	Regulatory	8/14	–
2567*; 2568*	Competence operon	3/11; 5/13	*25860* (TTA) *25870 (–)
*2792* (*adpA*)	Major developmental regulator	14/14	*26930* (TTA)
3195*	Unknown	5/14	–
*3423*	Possible antisigma factor (AbaA-like)	12/14	*17120* (TTA)
3550* (widespread among actinobacteria)	Helicase	6/14	*35850* (–)
3919* (abaB*)	LysR-like regulator of antibiotic biosynthesis	7/13	–
3943* (rstP*)	LacI-like regulator	5/14	*40040* (–)
*4114* (widespread among actinobacteria)	Sporulation-associated protein (ankyrin-like repeats)	9/14	*43220* (–)
*4263*	LuxR-like regulator	8/11	–
*4395*	Hydroxylase	12/14	–
*5203*		6/14	*49090*
*5460*	Possible antisigma factor (AbaA-like)	6/14	*50850* (TTA)
*5495*	Cyclic di-GMP cyclase/phosphodiesterase	7/14	*51100* (TTA)
*5970*		6/13	*26520*
6156*	Possible antisigma factor (AbaA-like)	5/14	*19600* (–)
6245*	Unknown	5/13	–
*6476*	Unknown	7/14	–
*6681*(*amfC*); 6685* (amfR*)	SapB biosynthetic enzyme; regulator of SapB biosynthesis	2/12; 7/12	–
*7251*	Possible phosphotransferase	10/12	*16190*
*7465* (*cvnC13*)	Component of conservon	9/12	*37840* (TTA)

* Throughout, examples included had a TTA codon in at least five genomes other than *S. coelicolor* (arbitrarily chosen as a level likely to indicate adaptive value). The table includes gene clusters likely to share a physiological role, selected because of the frequent occurrence of a TTA codon in one or another gene of the cluster.

### Previously unnoticed aspects of the occurrence of conserved TTA codons

Earlier analyses had indicated that most of the *S. coelicolor* TTA-containing genes were absent from the few other *Streptomyces* genomes then available, and where the genes were conserved, the TTA codons often were not (Li *et al*., 2007; Chater & Chandra, 2008). With the availability of more genome sequences, it became possible to make a more sensitive search for orthologues of the 147 *S. coelicolor* TTA-containing genes (or in some cases gene clusters). We identified 19 that were widespread and frequently TTA-containing in 13 other *Streptomyces* genomes (Table 2). In addition, a further 10 genes or gene clusters frequently had TTA codons, even though their *S. coelicolor* orthologues were TTA-free (asterisks in Table 2). As 27/29 of the TTA-containing genes/clusters were found only in *Streptomycineae*, we infer that these genes and their TTA codons have adaptive value to streptomycetes and not to other actinobacteria. As shown in Table 2, about half of these genes encode proteins likely to be closely implicated in gene regulation or signal transduction, although their targets are mostly unknown. They include five conserved paralogues of genes found in the *whiJ* cluster (see below for further discussion) and gene sets for highly modified oligopeptides that contribute significantly to the ability of aerial hyphae to grow into the air (such as SapB, also discussed below).

### Evolution of the multigene *whiJ* system, which represses development, and its abundant paralogues

**Fig. 7:**
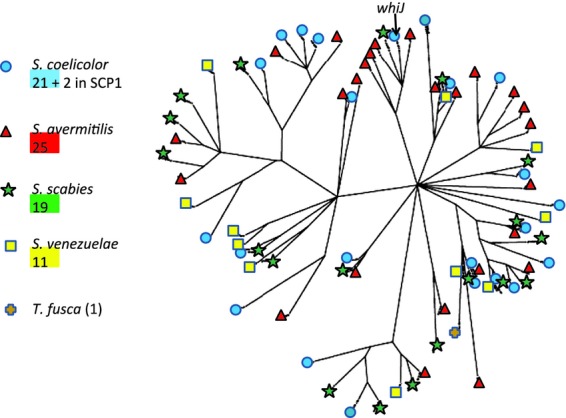
Phylogenetic analysis of WhiJ and its paralogues in four streptomycetes and another complex actinomycete. Genes encoding WhiJ paralogues were identified by probing translated gene products of the genomes of four streptomycetes [*S. coelicolor* A3(2) (blue circles); *S. avermitilis* (red triangles); *S. scabies* (green stars); *S. venezuelae* (yellow squares)] and *Thermobifida fusca* (brown crosses). The tree represents a phylogenetic analysis using PHYLIP (Felsenstein, 1989, 2005).

The central feature of the complex *whiJ* locus of *S. coelicolor* is the *whiJ* gene (SCO4543) for a deduced DNA-binding protein (Gehring *et al*., 2000; Ainsa *et al*., 2010). Most of the 24 paralogues of *whiJ* in the *S. coelicolor* chromosome are associated with one or both of two kinds of immediately neighbouring genes, one kind encoding very small DNA-binding proteins (i.e. like SCO4542) and the other encoding proteins with features like antisigma factors (e.g. SCO4544) (Gehring *et al*., 2000; Ainsa *et al*., 2010). *whiJ*-like genes are widely present in complex actinobacteria, but they are absent from morphologically simple ones (corynebacteria, mycobacteria, rhodococci, propionibacteria and micrococci except *Beutenbergia* and *Intrasporangium*) and from nonactinobacterial bacteria. These genes are often clustered with one or both types of *whiJ-*associated genes. Most mycelial actinomycetes have two or three WhiJ paralogues, but *K. setae* has five, and all streptomycetes have more than 10, sometimes more than 20. Phylogenetic analysis of WhiJ paralogues from four well-studied streptomycetes is shown in Fig. 7. The branching pattern is consistent with underlying sequential gene duplication events in an early progenitor of the four streptomycetes, followed by lineage-specific further divergence and duplication events. Phylogenetic analyses of the two *whiJ*-associated gene families gave broadly similar patterns, supportive of the idea that the genes in each cluster co-evolved (results not shown). WhiJ paralogues in *S. coelicolor* vary considerably in their conservation in other organisms. One (*SCO3421*) was present in nearly all complex actinomycetes, and the adjacent gene encoding a likely antisigma factor nearly always contained a TTA codon in *Streptomycineae* (but in no other groups). Another present in all *Streptomycineae* (*SCO4441*) was also widespread among other complex actinomycetes. Four others were found in all or nearly all *Streptomycineae* but no other groups (*SCO1242*, *1979*, *2513*, *6236*), among which a TTA was present in *SCO1242* and in the antisigma factor gene next to *SCO1979*. Future studies might profitably focus on these six relatively long-established clusters. Twelve other *S. coelicolor whiJ*-like genes were represented in around half of streptomycetes (SCO2381*, *2865*, 2869*, 3365*, *4176*, *4301*, *4678*, *4998*, *6129*, *6537*, 6629*, *7579*) [asterisks indicate occurrence also in some other complex actinomycete(s)]; while seven others were found in four or fewer streptomycetes (*SCO0704*, *2246*, *2253*, *4543*, *5125*, *6003*, *7615*). TTA codons were not associated with any of the last 19 genes mentioned or their associated genes.

Certain mutations in *whiJ* gave rise to a white-colony appearance caused by a deficiency in sporulation, although the complete deletion of *whiJ* had no obvious phenotypic consequences (Ainsa *et al*., 2010). A mutant lacking the *whiJ*-neighbouring gene *SCO4542*, encoding a predicted small DNA-binding protein, had a bald colony phenotype and overproduced the pigmented antibiotic actinorhodin. This phenotype was entirely suppressed by the co-deletion of *whiJ* itself. Putting these observations together, it was suggested that WhiJ acts mainly to repress reproductive development until a suitable signal has been perceived via the SCO4542 DNA-binding protein, which would then directly interact with WhiJ to relieve repression (Ainsa *et al*., 2010). It is thought that WhiJ mediates its effects both on the emergence of aerial hyphae and, separately, on their further differentiation into spore chains. There is no information about the direct or indirect targets of WhiJ regulation or about the role of the antisigma-like protein (SCO4544).

The apparently repressing action of the *whiJ* locus raises the possibility that some or all of its paralogues may also act as developmental brakes. If so, it may be that during ‘normal’ colony development on laboratory media, these brakes are all off – in other words, all relevant checkpoints have been passed, and the WhiJ-like proteins are not repressing their target genes. The acquisition of additional clusters would presumably confer species-specific environmental adaptations. As different streptomycetes vary in their ability to develop normally on different media, it is possible that this (partly) reflects differences in the complement of WhiJ-like signal transduction cascades. The strikingly reduced number of paralogues in *S. venezuelae* (one of several streptomycetes that lack a cluster orthologous to *whiJ* itself) may underpin the ability of *S. venezuelae* to sporulate exceptionally readily and comprehensively even in submerged culture, which has led to its adoption as a model system for development (Flärdh & Buttner, 2009; Bibb *et al*., 2012). Like the *wbl* genes described earlier, *whiJ*-like clusters are also found in plasmids (Bentley *et al*., 2004), permitting horizontal transfer. Interestingly, one of the ‘classical’ *bld* genes, *bldB*, encodes a diverged member of the SCO4542 family, but is an ‘orphan’ lacking neighbouring *whiJ-* or *SCO4544*-like genes. It is curious that *bldB* is the only classical *bld* gene to be confined to, yet universal among, streptomycetes (Fig. 3). We speculate that the bald phenotype of *bldB* mutants could imply a promiscuous interaction of BldB with WhiJ-like proteins encoded elsewhere in the genome and that this may be connected with the large numbers of such proteins found in streptomycetes.

## Special features of actinobacterial cell biology have contributed to the evolution of developmental complexity in *Streptomyces*

Cell growth and division in actinobacteria were recently thoughtfully reviewed by McCormick & Flärdh (2012) and Letek *et al*. (2012). Here, we consider the part played in these processes by conserved actinobacterial proteins, including some of the actinobacterial signature proteins in Table 1.

### The origins of mycelial growth: actinobacteria are unusual in predominantly using polar growth

At least in streptomycetes, corynebacteria and mycobacteria, cells grow by the insertion of peptidoglycan precursors at cell poles, guided by large, pole-located complexes of a coiled-coil-containing protein, DivIVA (Flärdh, 2003; Flärdh, 2010; Letek *et al*., 2008). The actinobacterial *divIVA* gene is nearly always located immediately next to an actinobacterial signature gene encoding a small probable membrane protein (*SCO2078*, Table 1a). It is an interesting possibility that this protein plays a part in the adaptation of DivIVA to polar growth in actinobacteria.

In *Bacillus subtilis* and other rod-shaped firmicutes (nonactinobacterial Gram-positive bacteria), DivIVA has a different role: it is involved in selection of the division site midway between opposite cell poles. Nevertheless, in such firmicutes, DivIVA is located at both poles, at least partly because of an affinity for concave membrane surfaces (Strahl & Hamoen, 2012). In these organisms, DivIVA binds the cell-division-inhibitory MinJDC protein complex – a mechanism that ensures that the cell centre contains the lowest concentration of Min proteins, so that cell division is medial. At firmicute cell division, DivIVA accumulates at the nascent septum, and upon cell separation, the new and old poles have similar amounts of DivIVA (Bramkamp & van Baarle, 2009).

In contrast, DivIVA does not accumulate rapidly at nascent septa in rod-shaped actinobacteria such as corynebacteria or mycobacteria, so newborn cells have an intrinsic asymmetry with respect to polar DivIVA complexes. In *M. tuberculosis,* the lag in formation of a full-sized DivIVA complex at the new pole is in fact very long – it is comparable with the interval between cell divisions, as shown by fluorescence microscopy of DivIVA::GFP fusions (Kang *et al*., 2008). This may reflect the complexity of the tip-organising complex (‘polarisome’: Hempel *et al*., 2012) that assembles round DivIVA and includes cytoskeletal elements and the cell wall biosynthetic apparatus (Holmes *et al*., 2013). The asymmetry in DivIVA distribution underpins a striking asymmetry in cell division observed by live-cell imaging of mycobacterial cells confined to microfluidic chambers (Aldridge *et al*., 2012): cells grow at just one pole, which is inherited stably; the newborn cells that lack an active pole take significant time to form one, and cell division depends on time rather than cell dimensions. As a result, the population of progeny cells in a very young microcolony is physiologically heterogeneous, different cell types even differing in their patterns of sensitivity to antibiotics (Aldridge *et al*., 2012).

**Fig. 8:**
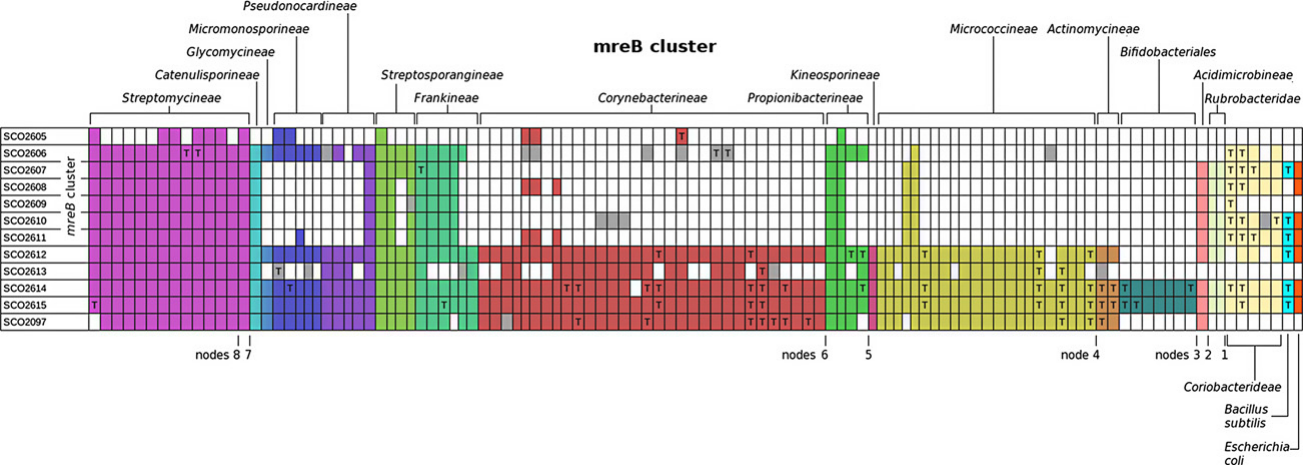
The *mre* gene cluster is absent from most simple actinobacteria. The reciprocal blastp best-hit tabulation includes the region from *SCO2605* to *SCO2615*. The numbered nodes refer to Fig. 2. See Fig. 3 legend and text for further details. The *mre* gene (*SCO2611*) is part of a cluster (*SCO2607*-*2611*) present in all streptomycetes and morphologically complex actinomycetes, but absent from nearly all mycobacteria and corynebacteria (rust red), and from members of the *Micrococcineae* (olive yellow), *Bifidobacteriales* (dark green) and *Rubrobacterideae* (brown). Interestingly, the adjacent gene *SCO2606* (encoding a likely radical SAM enzyme related to those involved in tRNA methylation) shows a very similar distribution. The Figure also shows the distribution of hits to the MreB-associated actinobacterial signature protein SCO2097 (Kleinschnitz *et al*., 2011).

Comparative genomic analysis suggests that DivIVA-mediated apical growth is typical of actinobacteria, as, on one hand, *divIVA* is universally conserved (always located close to the cluster containing *ftsZ* and other genes concerned with division and cell wall biosynthesis); while, on the other hand, the *mre* gene cluster (which mediates lateral cell wall growth in nonactinobacterial rod-shaped bacteria including *E. coli* and *B. subtilis*) is absent from nearly all rod-shaped or coccal actinobacterial genera originating from nodes 3 and 4 of Fig. 2 (Fig. 8). Actinobacteria on the deepest branches (nodes 1 and 2) do have the *mre* cluster, so it is not possible to infer which growth mode they might use. Although coccal actinobacteria also possess DivIVA, they may possibly grow in a DivIVA/MreB-independent manner, as in staphylococci: there, peptidoglycan growth is confined to the septum, which becomes remodelled from a circular to a hemispherical form during cell separation (Touhami *et al*., 2004).

What are the possible adaptive benefits of the two known growth modes of rod-shaped bacteria? The lateral wall growth of rod-shaped firmicutes may permit more efficient population growth, because the near-symmetry of growth and division allows both daughter cells to progress equally rapidly to subsequent divisions. In contrast, the asymmetry implicit in polar growth of rod-shaped actinobacteria has the potential to improve population resilience, because daughter cells have significantly different physiology, including different susceptibilities to some antibiotics (Aldridge *et al*., 2012). In the event of predivisional actinobacterial cells with three or more compartments (an apparent example of this may be seen in Fig. 5A of Singh *et al*., 2013), the tip-less compartments would have only nongrowing wall on their surface, with high levels of cross-linking. This might have enhanced survival value during exposure in the natural environment to physical stress, chemical or enzymatic attack of the wall itself, chemical or biochemical poisons (such as antibiotics produced by neighbouring organisms) and attack by bacteriophages. This increased resistance may have been a driving force for the evolution of mycelial growth.

A key requirement for the evolution of mycelial growth is a mechanism for cellular branching. This has become clarified by the discovery that, as a *Streptomyces* tip extends, it acquires increasing amounts of DivIVA (perhaps this is in some proportion to the number of genome copies in the tip compartment) and eventually splits, part remaining at the tip and part adhering to the lateral wall, which is thereby marked as a position of future branch emergence (Hempel *et al*., 2008; Flärdh *et al*., 2012). In a manner reminiscent of the situation already described for new poles in mycobacteria, branch emergence is not usually immediate, perhaps because the incipient polarisome has to be built up to some critical mass and/or organisation. However, new mycobacterial poles must be nucleated with DivIVA *de novo*, whereas mycelial branches are nucleated by the residue of a split polarisome, which may be a more avid target for DivIVA than septa.

*Streptomyces* polarisome splitting requires the activity of a serine/threonine protein kinase, AfsK, which phosphorylates DivIVA (Hempel *et al*., 2012). AfsK orthologues are present only in streptomycetes and *K. setae*, with a very weak hit also in *Catenulospora acidiphila*. How do other mycelial actinomycetes control branching? Most probably, by the action of other protein kinases on DivIVA – even in the nonbranching mycobacteria, DivIVA (=Wag31) is subject to phosphorylation during cell growth, but by serine/threonine protein kinases different from AfsK (Jani *et al*., 2010).

**Fig. 9:**
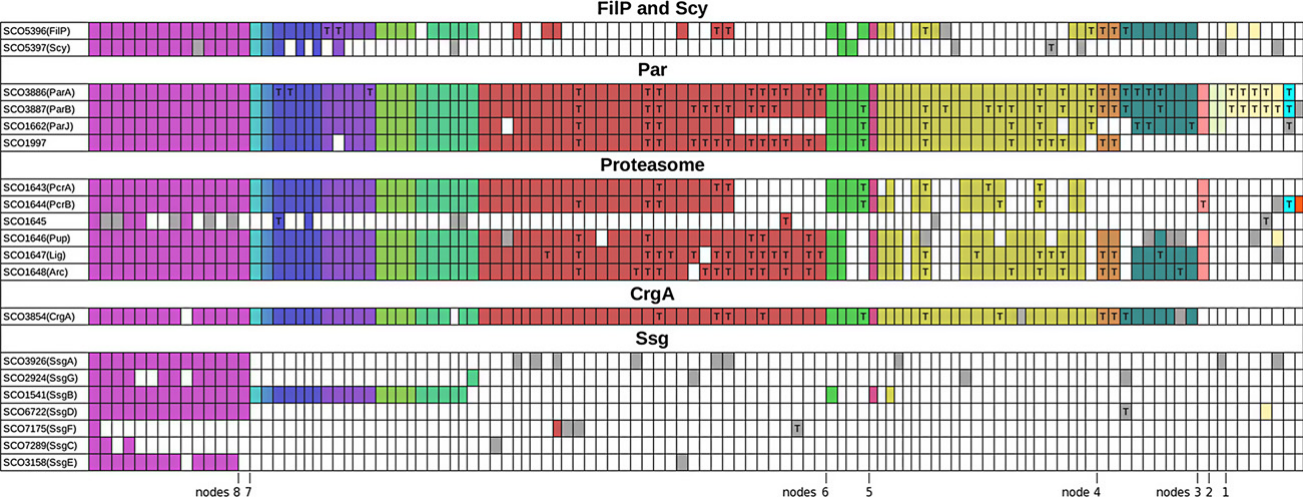
Distribution of some actinobacteria-specific cytoskeletal and related proteins. See Fig. 3 for further explanation of this reciprocal blastp best-hit analysis.

To achieve full mechanical strength, hyphae require FilP, a cytoskeletal coiled-coil protein that forms filaments along the hyphae (Bagchi *et al*., 2008). Orthologues of FilP appear to be very widespread among actinobacteria, including many morphologically simple organisms that diverged from the *Streptomyces* line early in evolution (e.g. bifidobacteria), but FilP is absent from coccal organisms, with the exception of *Kineococcus radiodurans* (Fig. 9). Thus, FilP is an ancient protein that may be important in generating resilient cylinders from the hemispherical nascent peptidoglycan emanating from poles. In streptomycetes, this involves a direct interaction with DivIVA (Fuchino *et al*., 2013). However, FilP is also apparently absent from corynebacteria and most mycobacteria. Possibly some other protein substitutes for it, or the sequence divergence of FilP in these organisms may be too great for identification by reciprocal blastp best-hit analysis.

### Ancient special features of actinobacterial cell division underpin the conversion of aerial hyphae into spores in *Streptomyces*

Surprisingly, little is understood about cell division in *Streptomyces* vegetative hyphae (Jakimowicz & van Wezel, 2012). Vegetative septa do not lead to cell separation, unlike their counterparts in nonmycelial actinobacteria. They are seldom found close to hyphal tips, and their positioning does not seem to conform to any recognisable pattern. A mycelium (albeit a relatively insubstantial one) can even be formed in the absence of septa, for example in an *ftsZ* mutant (McCormick *et al*., 1994; McCormick, 2009). Likewise, the mutational inactivation of chromosome partitioning functions ParA and ParB, or of the FtsK DNA translocase, has no effect on mycelial growth (Kim *et al*., 2000; Jakimowicz *et al*., 2005a; Jakimowicz *et al*., 2007; Wang *et al*., 2007; Ausmees *et al*., 2007; Dedrick *et al*., 2009). Effects of these mutations become apparent only during sporulation, when a kind of cell division more closely resembling that of unicellular organisms takes place. Here, we follow this parallel in a consideration of five partially overlapping aspects: (1) the transition from tip growth of an aerial hypha to sporulation septation; (2) the FtsZ-centred divisome; (3) partitioning of chromosomes into spores; (4) cell wall remodelling and thickening; and (5) spore germination.

**(1)** At the tips of aerial hyphae, a very long coiled-coil protein, Scy (Walshaw *et al*., 2010), interacts with two other coiled-coil proteins: DivIVA, and the FilP protein discussed in the preceding section. Scy is needed for properly organised sporulation septation (Holmes *et al*., 2013). Scy appears to be less widespread among actinobacteria than FilP, but its primary sequence is more prone to divergence (often a problem with comparative genomics of coiled-coil proteins), so reciprocal best-hit blastp analysis is less reliable. However, undoubted orthologues are present in some of the organisms emanating from node 4 onwards in Fig. 2 (Fig. 9). The authenticity of these hits is firm, because they are nearly all encoded by genes immediately next to *filP*. Most of the Scy-containing organisms show some propensity for mycelial growth. We tentatively suggest that, even in some simple actinobacteria, FilP may be involved not only in tip organisation, but also in communication between the tip and the establishment of septa, and that Scy modulates this communication in certain cell types of more complex actinobacteria, especially during reproductive fragmentation (including sporulation).

**(2)** In order for sporulation septation to take place, large amounts of the proteins making up the machinery of cell division must be made available. For *ftsZ* and *parAB*, this is achieved at least in part by the use of very strong sporulation-specific promoters, in addition to weaker ones used during vegetative growth. One of the most important roles of the Whi proteins discussed earlier is to cause such overexpression, in at least one case, directly (WhiA and the *parAB* promoter, as discussed earlier: Kaiser & Stoddard, 2011; Jakimowicz *et al*., 2006). Importantly, the activation of the sporulation-specific promoter of *ftsZ* is still not understood, although it is known that overexpression of *ftsZ* can partially suppress the phenotypes of all the *whi* mutants (Willemse *et al*., 2012) and that BldD represses the sporulation-specific promoter of *ftsZ* (den Hengst *et al*., 2010).

FtsZ of streptomycetes has special features dedicated to its role in sporulation, inasmuch as certain *ftsZ* mutations eliminate sporulation septation without eliminating vegetative septation (Grantcharova *et al*., 2003; Wasserstrom *et al*., 2013). The cell division apparatus (divisome) of actinobacteria also includes a form of DivIC so different from that of other bacteria that it was initially considered to be one of the actinobacterial signature proteins (Gao *et al*., 2006), until it was found by Bennett *et al*. (2007) to share 23/90 of the residues of conserved domain sequence 21 (pfam04977) of DivIC. (Gao & Gupta (2012) later removed this protein from their list of signature proteins because of its ‘presence in some other bacterial groups’.) The conserved domain 21 comprises a membrane-spanning segment and a C-terminal coiled-coil region located outside the cell membrane. The part of DivIC that is in the cytoplasm bears no apparent relatedness to DivIC in other bacteria. In other bacteria, DivIC cooperates with another membrane protein, FtsL, and the *S. coelicolor* protein likewise interacts with FtsL in a complex that contributes to both vegetative and sporulation septation. Mutants in *divIC* or/and *ftsL* are particularly affected in sporulation septation, forming many incomplete and asymmetrical septal ingrowths in aerial hyphal apical compartments of colonies grown on high osmolarity medium (Bennett *et al*., 2007). The primary amino acid sequence divergence between nonactinobacterial and actinobacterial DivIC proteins suggests co-evolution with some other cell division differences between organisms.

A remarkable feature of cell division in streptomycetes that appears specially significant for sporulation septation is the use of specialised actinobacteria-specific proteins (‘SALPs’, SsgA-like proteins) to determine the locations at which cell division and/or changes in cell wall structure will take place (Noens *et al*., 2005, 2007). Thus, SsgA is present in spores at the positions from which germ tubes will emerge, then at the growing tips of the apical compartments of aerial hyphae and then at the positions at which sporulation septation will take place (Noens *et al*., 2007), while the paralogous SsgB is more developmentally specific, appearing to be recruited by SsgA to the future sites of sporulation septation, where it forms a circumferential ring inside the cell membrane and directly recruits FtsZ (Willemse *et al*., 2011). SsgA and SsgB are present in all streptomycetes, as is SsgD, which is required for spore wall integrity (Fig. 9; Noens *et al*., 2005). Two other SALPs of *S. coelicolor* are present in some, but not all, other species: SsgE plays a part in the separation of spores, and SsgG is needed for the properly regular formation of sporulation septa (Noens *et al*., 2005). The other two *S. coelicolor* SALPs are absent from most species: SsgC, affecting the regularity of sporulation septation and partitioning of DNA into prespore compartments; and SsgF, affecting spore separation (Noens *et al*., 2005). It is not known whether other paralogues compensate for the absence of SsgC and SsgF in streptomycetes lacking them. Reciprocal blastp analysis with SsgB gave convincing hits with most developmentally complex actinomycetes and with *Cellulomonas flavigena* and *Kineococcus radiodurans*, but no other Ssg protein gave meaningful hits outside of *Streptomycetaceae*. It is likely that the acquisition of SsgB by an early actinomycete (before node 4 of Fig. 2) was a key to the later evolution of sporulation septation, but SALPs have been entirely lost independently from several subsequent branches.

The near-universal actinobacterial protein CrgA (Table 1, Fig. 9) has been studied in two streptomycetes (Del Sol *et al*., 2003, 2006) and in *M. tuberculosis* and *M. smegmatis* (Plocinski *et al*., 2011, 2012). It is a small protein with a C-terminal transmembrane domain. In *M. tuberculosis*, CrgA is abundant and interacts directly with cell division proteins (FtsZ, FtsQ, FtsI and PBPA) and with a newly characterised membrane protein, CwsA, that in turn interacts with the mycobacterial DivIVA orthologue Wag31 (Plocinski *et al*., 2012). It has been suggested that one role of CrgA in mycobacteria is to promote and/or stabilise FtsI localisation, facilitating septum formation (Plocinski *et al*., 2011) and helping to coordinate septal and polar peptidoglycan synthesis with FtsZ-ring assembly (Plocinski *et al*., 2012).

In streptomycetes, CrgA is considered to coordinate sporulation septation with hyphal growth, although it does not localise either to growing tips or to the sites of septation (Del Sol *et al*., 2006). This failure to co-localise with DivIVA is consistent with the absence of an obvious CwsA-like protein in *S. coelicolor*. The overexpression of CrgA inhibits septation (Del Sol *et al*., 2006), an effect that could be a secondary consequence of the over-occupation by CrgA of its various interaction partners. Interestingly, *crgA* mutants of streptomycetes do not always have the same phenotype: an *S. coelicolor* mutant shows premature production of spores that are slightly aberrant, but in the *S. avermitilis* mutant, sporulation septation does not take place (Del Sol *et al*., 2003).

**(3)** Actinobacteria all possess the ParABS partitioning system common to the majority of bacteria (but absent from *E. coli*). In this system, the ParB DNA-binding protein associates with newly replicated ParS sites located close to the chromosomal origin of replication (*oriC*), the two complexes being recognised by the ATPase motor protein ParA, which drives them apart (Toro & Shapiro 2010). The ParABS system has been shown to be important for the reliable segregation of chromosomes of corynebacteria and mycobacteria during normal growth, with ParB, ParA and Wag31 (= DivIVA) interacting in all pairwise combinations, presumably allowing coupling of polarisome activity to segregation (Donovan *et al*., 2010, 2012; Ginda *et al*., 2013). In these organisms, the Par proteins are predominantly associated with the cell pole (Donovan *et al*., 2010, 2012; Ginda *et al*., 2013), although transient localisation at the corynebacterial division septum is also seen for ParB, which appears to interact directly with FtsZ (Donovan *et al*., 2010). Thus, even in morphologically simple actinomycetes, the ParAB system provides a system of interplay between the growing pole, the septum and the chromosomal *oriC*. It appears that this has evolved to coordinate the more complex process of *Streptomyces* sporulation septation (Kim *et al*., 2000; Jakimowicz *et al*., 2005a, 2007).

Although the mechanism coordinating cessation of growth with the cessation of DNA replication and the initiation of chromosome partitioning in sporulating aerial hyphae of streptomycetes is not understood, a possible clue comes from vegetatively growing *B. subtilis*, in which there is interplay of the replication initiator protein DnaA with ParB (=Spo0J) and ParA (=Soj). DnaA is strongly inhibited by Soj monomers, but stimulated by ATP-dependent Soj dimers. The Soj monomers are generated from Soj/Spo0A complexes, simultaneously freeing Spo0A to form large partitioning complexes with the chromosomal *oriC* region (Scholefield *et al*., 2011). In these ParB/*oriC* complexes, ParB interferes with access of DnaA to *oriC*. During *Streptomyces* sporulation, ParB forms large complexes with the *oriC* region that probably have the same effect (Jakimowicz *et al*., 2002). Formation of these ParB complexes requires a shift in the behaviour of ParA, which is tip-associated while aerial hyphae are extending, but forms apparent helical filaments along the hyphae when growth stops (Jakimowicz *et al*., 2007). The ParA filaments dissociate immediately before sporulation septation and chromosome partitioning take place; and chromosome partitioning into prespore compartments is markedly irregular in the absence of ParA filaments or ParB (Jakimowicz *et al*., 2007). Dissociation involves interaction of ParA filaments with SCO1662 protein, one of two similar proteins (the other, SCO1997, shows near end-to-end alignment with SCO1662) that are among the ‘universally’ conserved actinobacterial proteins (Table 1A; Gao *et al*., 2006; Ditkowski *et al*., 2010). Like ParA, SCO1662 protein, renamed ParJ, is important for accurate chromosome partitioning into prespore compartments (Ditkowski *et al*., 2010). Actinobacteria lacking ParJ possess its SCO1997-like paralogue (Fig. 9). By analogy with *B. subtilis*, the action of ParJ in causing ParA polymers to dissociate may lead to inhibition of DnaA, reinforcing the repression of further DNA replication (but there is no published evidence of a ParA-DnaA interaction in any actinobacteria). As these proteins are all widespread in actinobacteria, such a mechanism could have ancient roots (Fig. 9).

Structures have been determined for the ParJ orthologue of *S. avermitilis* (Chang *et al*., 2010) and for SCO1997 protein (Gao *et al*., 2009) and its equivalents from *Corynebacterium glutamicum* and *M. tuberculosis* (Zhang *et al*., 2007; Graña *et al*., 2009). Although ParJ and SCO1997 proteins are only 28% identical to each other at the amino acid sequence level, both are structurally related to the PAC2 family of proteins (Gao & Gupta, 2012), which in eukaryotes are chaperones for the assembly of 20S proteasomes. Interestingly, although proteasomes are nearly universal among actinobacteria, they are absent from nearly all other bacteria (Striebel *et al*., 2013; Fig. 9) [proteasome-associated proteins were not listed among the actinobacterial signature proteins (Gao *et al*., 2006), probably because they happen to have been absent from two of the four organisms surveyed by those authors]. It therefore seems worth studying whether proteasomes also have an interface with this stage of the sporulation septation process. No genetic or cytological analysis of SCO1997 function has been reported, but all actinobacteria also possess a second *parA*-like gene (e.g. *SCO1772*, *parA2*: Fig. 9). In *C. glutamicum,* the product of this *parA* paralogue, named PldP, has been found to interact with ParB and to play a part in division site selection: PldP is predominantly found at developing septa (Donovan *et al*., 2010). Perhaps, in *S. coelicolor,* SCO1997 protein interacts with ParA2 to influence its aggregation state.

Although the Par system positions the *oriC* regions correctly, the parts of the sister chromosomes trailing behind the partitioning complex could potentially be guillotined by ingrowing septa, particularly in actinobacteria in which the septa are eccentrically located and no system of nucleoid occlusion of septation normally operates (although such a system may become operational in certain *Streptomyces* genotypes in which chromosome condensation is abnormal: Facey *et al*., 2009). However, as in most bacteria, FtsK-like DNA translocases (Reyes-Lamothe *et al*., 2012) are able to mobilise chromosome DNA through the septum as it closes, both in mycobacteria (Singh *et al*., 2013) and in sporulating aerial hyphae of *S. coelicolor* (Ausmees *et al*., 2007; Wang *et al*., 2007; Dedrick *et al*., 2009). FtsK orthologues were found in 97% of the actinobacteria considered in this article. In addition, a second weakly FtsK-like protein (SCO1416, SffA) is present in all streptomycetes, *Kitasataspora setae* and *Catenulospora acidiphila*, along with a small membrane protein SmeA encoded by the adjacent gene, *SCO1415*. SmeA and SffA are absent from all other actinobacteria, and both are specifically targeted to sporulation septa (Ausmees *et al*., 2007). Their roles are not known, but mutation of *smeA* causes aberrant sporulation septation (Ausmees *et al*., 2007).

The inclusion of a complete chromosome in each spore compartment is also aided by proteins that bring about nucleoid compaction. Facey *et al*. (2009) showed that *S. coelicolor* has three Dps proteins, which are all involved in sporulation-associated nucleoid partitioning and compaction as well as in the osmotic stress response. DpsB (SCO5756) is represented in most streptomycetes and many other actinobacteria, but DpsA (SCO0596) is less universally present, and DpsC (SCO1050) is absent from most. An intriguing interplay between the Dps proteins of *S. coelicolor*, indicated by surprising differences in the nucleoid compaction phenotypes of various single and double mutants (Facey *et al*., 2009), therefore does not seem to be generalisable among streptomycetes; and an extended analysis of the evolution of the three proteins (Facey *et al*., 2013) does not clarify this interplay. Contributions to spore nucleoid partitioning and compaction are also made by the Smc protein and its partner proteins ScpA and ScpB (Dedrick *et al*., 2009; Kois *et al*., 2009), and by sIHF (SCO1480: Yang *et al*., 2012; Swiercz *et al*., 2013). These proteins are also present in nearly all actinobacteria, SCO1480 being one of the actinobacteria-specific proteins listed by Gao *et al*. (2006) (Table 1). Another of the actinobacteria-specific proteins in Table 1, Lsr2 (SCO3375), is also a nucleoid-associated protein, functionally equivalent to H-NS of *E. coli* (Gordon *et al*., 2010), but it is not known whether Lsr2 has a developmental role in streptomycetes [all streptomycetes, and a few other actinomycetes, have a second Lsr2-like protein (e.g. SCO4076)]. The apparent absence of any effects of elimination of Dps, Smc or Scp proteins, and the relatively slight effect of sIHF elimination, on vegetative growth of *S. coelicolor*, are consistent with the idea that sporulation is a specialised version of a process that takes place even in unicellular actinobacteria, perhaps upon entry into stationary phase. The sIHF orthologues in *M. tuberculosis* and *M. smegmatis* appear to be essential (mIHF: Pedulla & Hatfull, 1998; Sassetti *et al*., 2003).

Another type of DNA-packaging protein, HupS, contributes to the final packaging of DNA in spores, although apparently not to the completion of partitioning (Salerno *et al*., 2009). HupS resembles HU proteins found throughout bacteria, but contains an extra domain peculiar to actinomycetes. HupS orthologues are absent from morphologically simple actinobacteria, including *Corynebacterium* spp. and most *Micrococcineae*, but they are present in nearly all mycelial actinobacteria and *Mycobacterium* spp. Interestingly, the *M. tuberculosis* orthologue (Hlp) is upregulated in anaerobically induced dormancy (Lee *et al*., 1998). As HupS contributes to resistance of spores to heat (Salerno *et al*., 2009), the first acquisition of its progenitor may have provided positive selection for the evolution of sporulation in actinobacteria.

**(4)** The remodelling and thickening of the cell wall of cylindrical, thin-walled prespore compartments, and their separation to generate near-spherical thick-walled spores, are carried out by a multiprotein complex organised by MreB and its paralogue Mbl (Heichlinger *et al*., 2011; Kleinschnitz *et al*., 2011). One of the proteins in this complex is the product of the actinomycete signature gene *SCO2097* (Kleinschnitz *et al*., 2011; Fig. 8). Thus, SCO2097 may be involved in the formation or rounding off of sporulation septa prior to separation, or the maturation of peptidoglycan at cell poles (which, during growth and cell division, contain nascent peptidoglycan that is not fully cross-linked), or the thickening of spore walls. These processes may also be needed in the formation of resting cells even of simple actinomycetes (and presumably also the ancestral ur-actinomycete): at least in *M. tuberculosis*, anaerobically grown resting cells not only have increased levels of the DNA-packaging protein Hpl (see preceding section) but also have thicker walls than aerobically grown cells (Cunningham & Spreadbury, 1998).

**(5)** Part of the biomass of incipent stationary phase cells is sacrificed to adaptations that improve survival against the ravages of time and environmental insults. These adaptations are most pronounced in spores, with their thickened cell walls, accumulation of stored reserves such as trehalose, and special packaging of nonreplicating DNA. Some of these features may act as barriers to growth, predicating the evolution of germination mechanisms, for example to permit breaking out from the thickened wall, and the casting off of DNA packaging. A widely conserved actinobacteria-specific protein family, represented in Gao *et al*. (2006) by the product of the *Mycobacterium leprae* gene *ML2030*, is made up of ‘resuscitation-promoting factors’ (RPF). The first example of an RPF was isolated from stationary phase culture fluids of *Micrococcus luteus*, where it was shown to greatly increase the number of colonies generated by plating out suspensions of stationary phase cells (Mukamolova *et al*., 1998). The closest relative of ML2030 in *S. coelicolor* is SCO3097, although the absence of local synteny makes it unclear whether they are true orthologues. The 70-aa Rpf domain of SCO3097 is also found in four other secreted proteins in *S. coelicolor*. Proteins possessing this domain are present in nearly all actinomycetes, often as a small series of paralogues. They are generally predicted to be secreted. The Rpf domain, which was predicted to be muralytic on bioinformatic grounds (Ravagnani *et al*., 2005), possesses a lysozyme-like fold (Cohen-Gonsaud *et al*., 2005; Ruggiero *et al*., 2009) and, at least in two RPFs, has demonstrable peptidoglycan hydrolase activity (Mukamolova *et al*., 2006; Haiser *et al*., 2009). Mutational analysis indicates considerable functional redundancy among RPFs in mycobacteria (reviewed by Kana & Mizrahi, 2010), but phenotypic effects were observed in *S. coelicolor* when *SCO3097* was disrupted: the mutant produced thin-walled, heat-sensitive, irregular spores in chains that tended not to separate as readily as wild-type spore chains and showed a modest delay in germination (Haiser *et al*., 2009).

Interestingly, RpfB and RpfE (but not RfpA, C or D) proteins of *M. tuberculosis* are found in a synergistically acting complex with another kind of peptidoglycan hydrolase (RipA; Hett *et al*., 2007, 2008) and appear to play dual roles in cell wall growth and septation on one hand, and emergence from dormancy on the other. Clearly, such hydrolytic activity must be highly controlled if cell wall integrity is to be retained. One mechanism for this control may involve the peptidoglycan biosynthetic enzyme PBP1, which interacts with the RpfB/RipA complex and inhibits its hydrolase activity, most likely by a RipA partner-switching mechanism, as RipA uses the same C-terminal 25 aa to interact with PBP1 and RpfB (Hett *et al*., 2010). In organisms exhibiting polar growth, septation requires the nongrowing (and presumably most extensively cross-linked) part of the cell wall to be re-established as a template for renewed synthesis, so Rpf proteins may be involved in this. RpfB and RipA localise to the septa of mycobacterial cells (Hett *et al*., 2007), while PBP1 localises mainly to cell poles but also to some septa (Hett *et al*., 2010).

Re-establishment of cell wall growth is also needed for the emergence of hyphal side branches. Could Rpf proteins and RipA homologues influence branching, including aerial branches, in *Streptomyces*? Streptomycetes have proteins modestly resembling RipA – the most similar, SCO4793, has near end-to-end *c*. 30% identity with RipA, but has an additional central segment. The evolutionary origin of the RPF domain and its interaction with RipA-like proteins may be hinted at by the finding that the ‘tape-measure’ proteins of many actinophages include both domains and, in at least one instance, can degrade the cell walls of stationary phase host bacteria (Piuri & Hatfull, 2006).

## Extracellular functions important for *Streptomyces* sporulation mostly entered the actinobacterial lineage at the time when morphological complexity emerged

*Streptomyces* aerial growth and sporulation depend on both nutritional and mechanical support. Some of the elements involved are extracellular and have been discussed in some detail (Chater *et al*., 2010). Here, we show that the acquisition of some of these elements coincided with the last node preceding the emergence of complex actinomycetes (Fig. 2), raising the possibility of causal connections.

### An extracellular protease cascade

One important source of nutrients for aerial growth is provided by lysis (sometimes referred to as ‘programmed cell death’: Manteca *et al*., 2007) of the part of the mycelium from which sporulating aerial hyphae emerge. Studies in *S. coelicolor* and several other species indicate that this lytic process involves an extracellular protease cascade (Chater *et al*., 2010). The cascade is held inactive by an extracellular protease inhibitor protein, such as Sti of *S. coelicolor* (Kim *et al*., 2008a). Using blastp reciprocal best hits as a guide, most *Streptomyces* genomes contain a *sti-*like gene, and such genes are also present in most developmentally complex actinomycetes (*Catenulosporineae*, *Glycomycineae*, *Micromonosporineae*, *Pseudonocardineae* and *Streptosporangineae*, but not *Frankineae*: Table 3). This suggests that extracellular protease cascades may have been significant in the evolution of actinomycete complexity. Among streptomycetes, the sequences of the protease inhibitors diverge more than most other conserved gene products, even though there is detectable synteny of the determinants (Table 3; see also Fig. 6). It is therefore possible that divergence of the different inhibitors may have contributed to speciation, with the inhibitors being species-specific. A similar argument was used earlier, in considering the antisigma factor RsbN.

**Table 3:** Occurrence of Sti-like protease inhibitor genes (*SCO0762*-like) in complex actinomycetes

Organism	Amino acid identity in blastp	Length of overlap	Local synteny
*Streptomyces lividans*	99%	100%	Yes
*Streptomyces avermitilis, griseoflavus, sviceus, viridochromogenes*	72–77%	*c*. 100%	Yes
*Streptomyces clavuligerus, griseus, hygroscopicus, pristinaespiralis, roseosporus, scabies, venezuelae*	50–66%	> 75%	*SCO0767* orthologue in most cases
*Streptomyces albus, Kitasatospora setae*	No reciprocal hit		
*Catenulispora*	37%	47%	No
*Stackebrandtia*	49%	60%	No
*Micromonospora aurantica, M*. sp. *L5*	45%	73%	No
*Actinosynnemma, Amycolatopsis mediterranea, Saccharomonospora viridis, Saccharopolyspora erythraea*	36–41%	61–80%	No

Sti has two activities – a general one against serine proteases and a specific one mediated via specialised C-terminal interaction domains on target proteases (P-domains: Kim *et al*., 2008a; Chater *et al*., 2010). Two P-domain proteases, SCO1355 (serine peptidase) and SCO5447 (neutral zinc metalloprotease), are present in *S. coelicolor*. A survey of the genomes in StrepDB (http://strepdb.streptomyces.org.uk) shows P-domains to be present typically in up to five proteins in any one streptomycete (Table 4). Although these proteins were mostly serine proteases, two examples of P-domain aminopeptidases were found. The six genomes surveyed all encoded other proteases closely similar to the P-domain-containing proteases, but lacking the C-terminal P-domain. This complicates consideration of the significance of the Sti-like protease inhibitors, as the proteases without P-domains would presumable bypass inhibition. Possibly, there are important differences in the regulation or substrate specificities of the proteases with and without P-domains. Future studies may address the question of how P-domains have been acquired or lost over evolutionary time.

**Table 4:** P-domain-containing proteins in six streptomycetes

*Streptomyces* species	P-domain-containing neutral zinc metalloendoproteases (orthologue)	P-domain-containing serine endo- /exopeptidases (orthologue)	P-domain-containing putative aminopeptidases
*S. coelicolor*	SCO5447*	**SCO1355**	–
*S. avermitilis*	SAV_2794*, 1037	–	–
*S. clavuligerus*	**SCLAV_4359**	–	–
*S. griseus*	**SGR_2095 (=** ***sgmA*****)**	**SGR_6802**, 2549, 918 (3 P-domains)	SGR_5809
*S. scabies*	SCAB27921*	20151, 89701	–
*S. venezuelae*	**SVEN_5109**	SVEN_4741, 6538, 6996,	SVEN_4288

* Adjacent gene encodes close paralogue without a P-domain.

Sti-mediated inhibition of the *S. coelicolor* protease cascade is released by specific cleavage of Sti by another protease, SCO5913 (Kim *et al*., 2008a,b). blastp analysis showed that orthologues of the Sti-degrading protease SCO5913 were absent from all species examined except *S. lividans*, a very close relative of *S. coelicolor* that has a nearly identical Sti determinant. Thus, the set of proteases involved in developmental proteolysis may differ in different streptomycetes.

Analogies with eukaryotic P-domain proteases suggest that, on their release from inhibition, *Streptomyces* P-domain proteases cleave, and thereby activate, pro-enzymes that then go on to take part in development. One such protein may be transglutaminase (TGase), which may be involved in cross-linking surface-located proteins (Zotzel *et al*., 2003a,b; Zhang *et al*., 2008b), although TGase is absent from some species including *S. coelicolor*. Among the substrates for *Streptomyces morbaraensis* TGase are three protease-inhibiting proteins, each having specificity for a different protease involved in TGase activation (Schmidt *et al*., 2008; Sarafeddinov *et al*., 2011). Not all Sti-like proteins from other streptomycetes are substrates for the TGase (Taguchi *et al*., 2000), perhaps providing a window into the unusually highly diverged phylogeny of Sti orthologues referred to above. In a further twist, it appears that in *Streptomyces hygroscopicus,* the action of TGase results in the incorporation of the surfactant Sti-like protein into the surface of aerial hyphae (Zhang *et al*., 2008a), which may contribute in some way to the exoskeleton that we describe next.

### Exoskeletal proteins

Physical support for aerial growth of streptomycetes is provided by various secreted amphipathic proteins (chaplins and rodlins) and peptides (SapB-like) that can assemble on the hyphal surface (Claessen *et al*., 2003, 2004; Elliot *et al*., 2003; Willey *et al*., 2006; Capstick *et al*., 2007, 2011). These may solve two problems – the breaching of the surface tension at an air–water interface when aerial hyphae emerge from a hydrated environment, and the provision of a hydrated extracellular compartment to aerial hyphae, permitting ‘normal’ cell physiology such as the generation of membrane potential to be sustained, as well as potentially providing a route for nutrients to reach the apical compartment (Wosten & Willey, 2000; Chater *et al*., 2010; Chater, 2011).

Chaplins typically occur in long and short forms, the long chaplins containing a C-terminal domain that is a substrate for attachment to the cell wall by the action of sortase enzymes. Chaplins assemble at air–water interfaces and on the surface of hyphae growing into the air (Claessen *et al*., 2003; Elliot *et al*., 2003; Elliot & Talbot, 2004). All *Streptomycetes* have several chaplins. *Streptomyces coelicolor* has eight, but none of these is represented in every streptomycetes: four (ChpB, C, D, E) are found in more than half of other species (Di Berardo *et al*., 2008); and ChpB and ChpD reciprocal blastp hits are present in the nearest known relatives of streptomycetes, *K. setae* and *Catenulispora acidiphila*. Further blastp analysis revealed that *K. setae* had four other short chaplins. When the *K. setae* chaplin complement was used in blastp reciprocal best-hit analysis, further genes for chaplin-like proteins were found in a few developmentally complex organisms that are more remotely related to streptomycetes (*Stackebrandtia nassauensis*, *Thermobifida fusca, Streptosporangium roseum* and *Nocardiopsis dassonvillei*) and even in the nonsporulating rod-coccus *Arthrobacter chlorophenolicus*. It has been shown that some chaplins also play a significant role in mycelial attachment to surfaces (De Jong *et al*., 2009b), a function that might conceivably have preceded their role in aerial growth and might explain their presence in organisms not known to exhibit aerial growth.

Chaplins can assemble into paired rodlet structures under the influence of rodlin proteins, of which there are two in *S. coelicolor*. Obvious rodlins were found only in streptomycetes, although very low-scoring reciprocal blastp best hits were present in a few other morphologically complex actinomycetes (*Thermomonospora curvata* and *Stackebrandtia nassauensis*). They appear to be lost readily, as *S. avermitilis*, *S. griseoflavus* and S. *hygroscopicus* lack rodlin genes.

The *S.coelicolor* modified oligopeptide SapB is a post-translationally processed product of the small gene *ramS* (*SCO6682*; Kodani *et al*., 2004). AmfS, the equivalent oligopeptide of *S. griseus*, has also been studied extensively (Ueda *et al*., 2002). Processing is carried out by the product of the adjacent gene *ramC* (*SCO6681*) and includes the generation of lanthionine bridges like those found in lantibiotics. Two adjacent transporter genes (*SCO6683*, *SCO6684*) are thought to be responsible for SapB export, and the cluster depends on the regulatory gene *ramR* (*SCO6685*). We found similar clusters in most streptomycetes (*S. hygroscopicus* was an exception) and sporadically among other morphologically complex genera (but *ramS* orthologues are sometimes missed in the reciprocal blastp analysis because these genes are very small). As already mentioned, most *Streptomyces amf*-like clusters contain a TTA codon either in the *ramR*-like gene or in the *ramC* orthologue. We found one occurrence of a *ramS*-like gene among simpler actinomycetes, in *Kribbella flavida*, but in this case, the orthologues of the rest of the *amf* cluster were scattered. The regulatory gene *ramR* was found only in streptomycetes.

As in the cases of Sti-like protease inhibitors and RsbN antisigma factors already discussed, apparent orthologues of the genes in the *amf* cluster are unusually diverged, which might suggest a role in speciation (Fig. 6). However, species specificity in the action of SapB-like proteins has not been reported, and a SapB-deficient mutant of *S. coelicolor* could be induced to undergo full aerial growth and sporulation by adding SapT, isolated from *Streptomyces tendae*, or a fungal hydrophobin, SC3 (Kodani *et al*., 2005).

### Pathways related to that for a *Streptomyces* spore wall pigment are present in other complex actinomycetes

At a late stage of sporulation, the spore wall is modified by the attachment of an aromatic spore pigment, which may be specified either by a type II polyketide biosynthetic gene cluster (the *whiE* cluster of *S. coelicolor*: Davis & Chater, 1990) or a type III polyketide synthase (e.g. in *S. griseus*: Funa *et al*., 1999, 2005). The process may be equivalent to lignification (although such speculation is untested). In the case of *whiE*, a cluster of eight genes *SCO5314-5321* is required (Davis & Chater, 1990). Paralogues of the core *whiE* polyketide synthase genes are frequently present in gene clusters for type II polyketide antibiotics, which might complicate the interpretation of reciprocal blastp analysis; but despite this, the occurrence in some non-*Streptomyces* genomes of several adjacent reciprocal hits to *whiE* genes makes a strong case for the presence of a *whiE* pathway. On this basis, a *whiE*-like pathway is present in species of *Catenulispora, Frankia, Micromonospora, Salinispora, Amycolatopsis, Saccharomonospora, Kitasatospora, Nocardiopsis* and *Streptosporangium* – all complex sporulating species. Phylogenetic trees of WhiE proteins were broadly congruent with the actinobacterial phylogeny (not shown). Most likely, therefore, the WhiE pathway was present in the last common ancestor of these organisms (node 6 of Fig. 2). Phylogenetic analysis showed that the polyketide synthases for aromatic polyketides such as actinorhodin and tetracyclines did not emerge from within the WhiE lineage (Metsa-Ketela *et al*., 2002), so the common ancestral synthase presumably pre-dated the evolution of complex actinomycetes.

## Conclusion – how development develops during evolution

At the start of this article, we raised several questions that might be addressed in the light of the information reviewed. Here, we return to these questions.

### What are the evolutionary origins of genes specifically important for *Streptomyces* development?

**Fig. 10:**
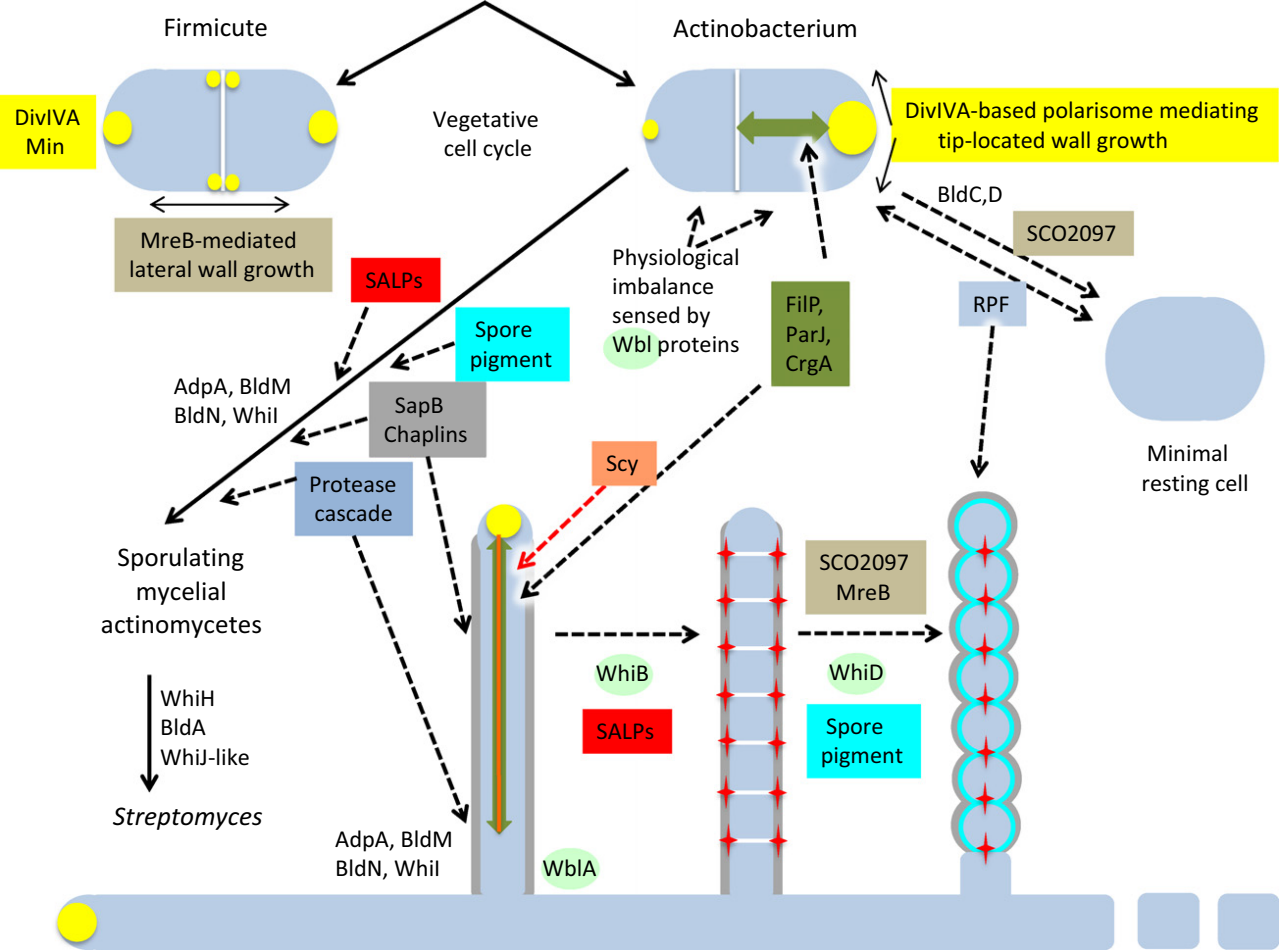
Acquisition and roles of actinobacteria-specific genes and processes during evolution. It is postulated that a difference in the location of cell wall growth, associated with the use of different central organising proteins (MreB or DivIVA) for peptidoglycan synthesis, was a key element in the early separation of the lines leading to firmicutes and actinobacteria. Polar growth may have been facilitated by the early acquisition of certain conserved cell-biological proteins (FilP, ParJ, CrgA) and proteasomes. Wbl proteins may have sensed the sudden difference in cell physiology consequent on asymmetrical division into a larger rapidly growing daughter cell and a smaller virtually nongrowing one. Entry of primitive *Actinobacteria* into stationary phase probably involved regulation by orthologues of BldD and BldC (as well as of actinobacteria-non-specific proteins such as WhiA and WhiG that are not shown). Tip growth potentiated the emergence of mycelial growth, which in turn predicated the need for some kind of fragmentation, which takes the form of sporulating aerial mycelium in *Streptomyces*. The stepwise acquisition of additional functions playing important roles in *Streptomyces* development is indicated. Proteins with structural or enzymatic roles are shown in coloured boxes, and their locations (or the locations of their products) are shown in corresponding colours. Regulatory proteins are unboxed. Wbl proteins are shown in pale green ovals.

A gene is considered specifically important for development if the relevant mutation has detectable phenotypic effects on development, but not on vegetative growth. Orthologues of many such genes were evidently present in early simple actinobacteria including some of the major developmental regulatory genes (though there are important exceptions). This suggests (in answer to another of the questions) that research focused on *Streptomyces* developmental biology is likely to provide clues about the cell biology of simpler actinobacteria, including pathogenic organisms, and vice versa. We have tried to summarise some of these reflections across taxa and time in Fig. 10.

### Are the mechanisms leading to sporulation widely homologous in phylogenetically diverse actinobacteria or did they evolve independently?

There is not a simple answer to this, but it does appear that there is a single underlying shared evolutionary pathway, involving both regulatory and cell-biological elements originating from the last common ancestor some 2.6 billion years ago (Battistuzzi *et al*., 2004), followed by the sequential addition of further elements. Only a few of the latter are specific to streptomycetes and/or their very closest relatives: in one such case, the role of AfsK in controlling DivIVA-mediated branch formation in *Streptomyces* is likely to be taken by different protein kinases in other mycelial organisms; and in another case, WhiH may provide little more than a modulating influence on the balance of components needed to maximise the efficiency of sporulation in the particular context of *Streptomyces* aerial hyphae. A very small number of developmental genes have been acquired so recently that they are specific to particular streptomycetes – the *whiJ* cluster is a good example of this.

### Does the developmental process contribute to speciation?

In this review, we have noted two kinds of species-associated diversity that might imply an interplay of development with speciation. One involves the richness and diversity of multiple paralogues of ‘*whiJ*-like’ clusters, which may imply differences in the sensitivity of development to environmental input (but we also note that the three types of protein encoded in these clusters are all predicted to be cytoplasmically located, so any such sensory role would probably require the independent uptake of soluble small molecules). On the other hand, the second kind of species-associated diversity involves unusually high divergence between orthologous extracellular proteins or proteins with a likely extracellular face. The examples highlighted are Sti-like protease inhibitors, SapB-like aerial growth-facilitating proteins and their biosynthetic enzymes, and the anti-sigma-BldN protein RsbN. These are all candidates for further evaluation as possible agents of speciation, although other explanations such as selection only for broad structural conservation may apply in some cases.

### Are today's simple actinobacterial species primitive or are they degenerate descendants of morphologically much more complex ancestors?

It is clear from our analysis that ancestral developmental genes have been lost in some phylogenetic branches (for example, FilP is absent from corynebacteria, and WhiG has been lost many times), showing that evolution does not always proceed in the direction ‘simple to complex’. Nevertheless, the actinobacteria diverging at very early nodes from the line leading to *Streptomyces* (Fig. 2) do have simple morphology and do lack some important developmental genes.

### What gave the ancestral ur-actinobacterium the potential for mycelial growth and aerial sporulation in its more modern descendants?

We have provided some circumstantial evidence and speculative discussion in support of the idea that DivIVA-mediated polar growth could have provided the platform for the evolution of mycelial growth and that actinobacteria-specific features of cell division may have contributed to the specialised processes by which aerial hyphae form chains of spores. However, some innovations, presumably acquired through horizontal gene transfer, were apparently very significant in providing the physical scaffolding for aerial growth (notably the chaplins and SapB-like proteins) and the specialised control of a proteolytic cascade that allows reuse of the substrate mycelium biomass to support aerial growth (Sti-like protease inhibitors), both of which appeared in the actinobacterial lineage at the earliest node giving rise to aerial mycelium.

### Can studies of the development of complex actinomycetes assist our understanding of the cell biology of their simpler cousins?

Orthologues of some of the proteins involved in hyphal growth and sporulation of streptomycetes, including several that were previously ‘function unknown’, are found in simple organisms that diverged early in the actinobacterial lineage. The *Streptomyces*-led breakthroughs in understanding how some such proteins influence hyphal polarity, and others sporulation septation or spore morphogenesis, may provide fertile testing ground for understanding the early evolutionary divergence of proto-actinobacteria from firmicutes and other simple bacteria.

### A new question: what is the meaning of disparities in the distribution of developmental genes?

A surprise emerging from this analysis was the occurrence of genes associated with developmental complexity in *Kineococcus radiodurans,* an apparently simple organism belonging to a phylogenetic group originating from a node that preceded those leading to well-defined complex groups. This raises two perplexing questions, and we have no answers to either of them: what functions do these genes fulfil in a simple coccus? And how is one to account for their phylogenetically inappropriate occurrence?

## Concluding comment

This review was stimulated by Gao and Gupta's search for actinobacterial signature proteins. We believe that many aspects of microbial behaviour and physiology can be illuminated by paying close attention both to ancient and less ancient taxon-specific proteins. Reciprocal blastp best-hit analysis such as is shown in Figs 3, 4, 8 and 9 has proved a considerable aid to thinking about the evolution and mechanisms of actinobacterial cell biology and developmental complexity, and access to the full tables at http://streptomyces.org.uk/actinoblast/ may prove useful in investigating other aspects of actinobacterial biology.
